# A Comparative Study of Variables Influencing Ischemic Injury in the Longa and Koizumi Methods of Intraluminal Filament Middle Cerebral Artery Occlusion in Mice

**DOI:** 10.1371/journal.pone.0148503

**Published:** 2016-02-12

**Authors:** Gary P. Morris, Amanda L. Wright, Richard P. Tan, Amadeus Gladbach, Lars M. Ittner, Bryce Vissel

**Affiliations:** 1 Neurodegenerative Disorders, Garvan Institute of Medical Research, Sydney, Australia; 2 Faculty of Medicine, University of New South Wales, Sydney, Australia; 3 Heart Research Institute, 2042 New South Wales, Sydney, Australia; 4 Dementia Research Unit, Department of Anatomy, School of Medical Sciences, Faculty of Medicine, University of New South Wales, Sydney, Australia; 5 Neuroscience Research Australia, Sydney, Australia; 6 Faculty of Science, University of Technology Sydney, Sydney, Australia; University of Naples Federico II, ITALY

## Abstract

The intraluminal filament model of middle cerebral artery occlusion (MCAO) in mice and rats has been plagued by inconsistency, owing in part to the multitude of variables requiring control. In this study we investigated the impact of several major variables on survival rate, lesion volume, neurological scores, cerebral blood flow (CBF) and body weight including filament width, time after reperfusion, occlusion time and the choice of surgical method. Using the Koizumi method, we found ischemic injury can be detected as early as 30 min after reperfusion, to a degree that is not statistically different from 24 h post-perfusion, using 2,3,5-Triphenyltetrazolium chloride (TTC) staining. We also found a distinct increase in total lesion volume with increasing occlusion time, with 30–45 min a critical time for the development of large, reproducible lesions. Furthermore, although we found no significant difference in total lesion volume generated by the Koizumi and Longa methods of MCAO, nor were survival rates appreciably different between the two at 4 h after reperfusion, the Longa method produces significantly greater reperfusion. Finally, we found no statistical evidence to support the exclusion of data from animals experiencing a CBF reduction of <70% in the MCA territory following MCAO, using laser-Doppler flowmetry. Instead we suggest the main usefulness of laser-Doppler flowmetry is for guiding filament placement and the identification of subarachnoid haemorrhages and premature reperfusion. In summary, this study provides detailed evaluation of the Koizumi method of intraluminal filament MCAO in mice and a direct comparison to the Longa method.

## Introduction

The most common human focal cerebral ischemia occurs due to thrombotic or embolic occlusion of the middle cerebral artery (MCA). Rodent models have therefore predominantly aimed to mimic MCA occlusion (MCAO), however no single rodent model fully recapitulates the features of human MCAO and each model has various advantages and disadvantages [1,2]. The intraluminal filament method developed by Koizumi et al., [3] and later modified by Longa et al., [4] has become a widespread model of choice for mimicking MCAO in rodents, due to the minimally invasive technique involved, and ability to allow reperfusion post-occlusion [5]. The later point is particularly important considering early restoration of blood supply is a major determinant of the severity of ischemic injury [6], reflected in the success of thrombolytic therapy following acute ischemic stroke in some individuals [7].

However, despite having been utilised for almost three decades, and many attempts to reduce variability [8,9], the intraluminal filament method still displays large inconsistency in the volume of ischemic lesions generated, both within studies and from lab to lab. For example, following similar protocols, different groups have obtained mean ischemic lesion volumes ranging from as much as 11.1–55.6% of the ipsilateral cortex, for a 30 min intraluminal thread occlusion in mice [2]. Dirnagl highlighted standard deviations in infarct volumes are as much as 40% of the mean in many studies [10]. These figures are indicative of the difficulty in generating robust and reproducible data from pre-clinical stroke studies.

Although simple in theory, the intraluminal filament method of MCAO requires practice to master the surgical skills necessary for success, and becomes more complex surgically when moving from a rat to a relatively smaller mouse. Furthermore, the complexity increases when considering the many variables shown to impact resulting ischemic lesion volume. These include the physical properties of the chosen embolus, such as the diameter and material [11,12], occlusion time [13], recovery time [14], temperature during and post-surgery [15], choice of anaesthetic [16], minor differences in surgical technique [8], experience of the surgeon [17], choice of animal (rat vs. mouse) [2], strain of animal [18], animal to animal variation in cerebrovasculature (such as the patency of the posterior communicating artery (PComA)) [19], sex [20] and age [21], among many others.

In both the Koizumi and Longa methods of intraluminal filament MCAO, one of the foremost causes of inconsistency derives from the choice of embolus (intraluminal filament), which can vary widely in thickness, length and in the material used to create it. A recent popular choice has been silicon coated silk sutures, which offer greater consistency in preparation compared to traditionally used homemade versions, such as heat-blunted sutures [11]. Considering the relatively recent introduction of these filaments, few comprehensive studies have been undertaken to determine the efficacy of silicone coated sutures to produce consistent lesions, or how often they may induce common side effects of the intraluminal filament surgery, such as subarachnoid haemorrhage (SAH), or high rates of premature death after reperfusion, which have in the past plagued the reliability of the method.

Secondly, despite the widespread use of both the Koizumi and Longa methods in rats and mice, the Longa method appears to have proliferated more widely throughout the literature. The use of mice is becoming more commonplace owing to the availability of many different genetically modified strains, and therefore it is important to compare these two methods to determine when and why each should be utilised in the mouse. There are two key differences between the methods; 1. The Longa method allows reperfusion via both central carotid arteries (CCA), as opposed to reperfusion via only one CCA in Koizumi’s method and 2. The Longa method traditionally employs filaments that only have a bulbous tip produced by heat-blunting sutures, whereas the Koizumi method initially utilised filaments coated with silicone for 5 mm at a consistent width [22].

In this study we have therefore compared three types of silicone coated filaments, varying in thickness and coating length, time after reperfusion, occlusion time and the efficacy of the Koizumi vs. the Longa method against various outcome measures including ipsilateral lesion volume, body weight loss, survival rates and neurological scores.

Finally, it has become commonplace to employ a tool to measure regional cerebral blood flow, such as laser-Doppler flowmetry, or synchrotron radiation angiography, to assist with confirmation of successful MCAO [23]. Data obtained from these recordings is often utilised to indicate SAH and premature reperfusion during occlusion, as well as being used to remove data on the basis of predetermined cut-off points for relative changes in CBF following MCAO. A commonly cited figure for exclusion of data is a <70% reduction in CBF following MCAO by an intraluminal filament. If measuring blood flow continuously throughout occlusion, or reapplying at a later time, these tools can also allow a measurement of ‘reperfusion’ following withdrawal of an embolus. In this study we examine the value of laser-Doppler measurement in determining SAH and premature reperfusion, and determine whether there is any valid statistical support for exclusion of data based on the ‘<70%’ alteration in CBF following MCAO in mice.

## Materials and Methods

### Animals

Male C57BL/6JAusb mice aged 8–13 weeks were obtained from Australian BioResources in New South Wales, Australia. Prior to surgery mice were housed in groups of 4–5 per cage and post-surgery mice were single housed, with *ad libitum* access to food and water. They were maintained on a 12 h light-dark cycle, with lights being switched on at 7.00 am. All surgeries were performed during light hours. All animal experiments were performed with the approval of the Garvan Institute and St. Vincent's Hospital Animal Ethics Committee, in accordance with National Health and Medical Research Council animal experimentation guidelines and the Australian Code of Practice for the Care and Use of Animals for Scientific Purposes (8^th^ edition, 2013).

### Ischemic model

#### Pre-surgery

**Fig 1:**
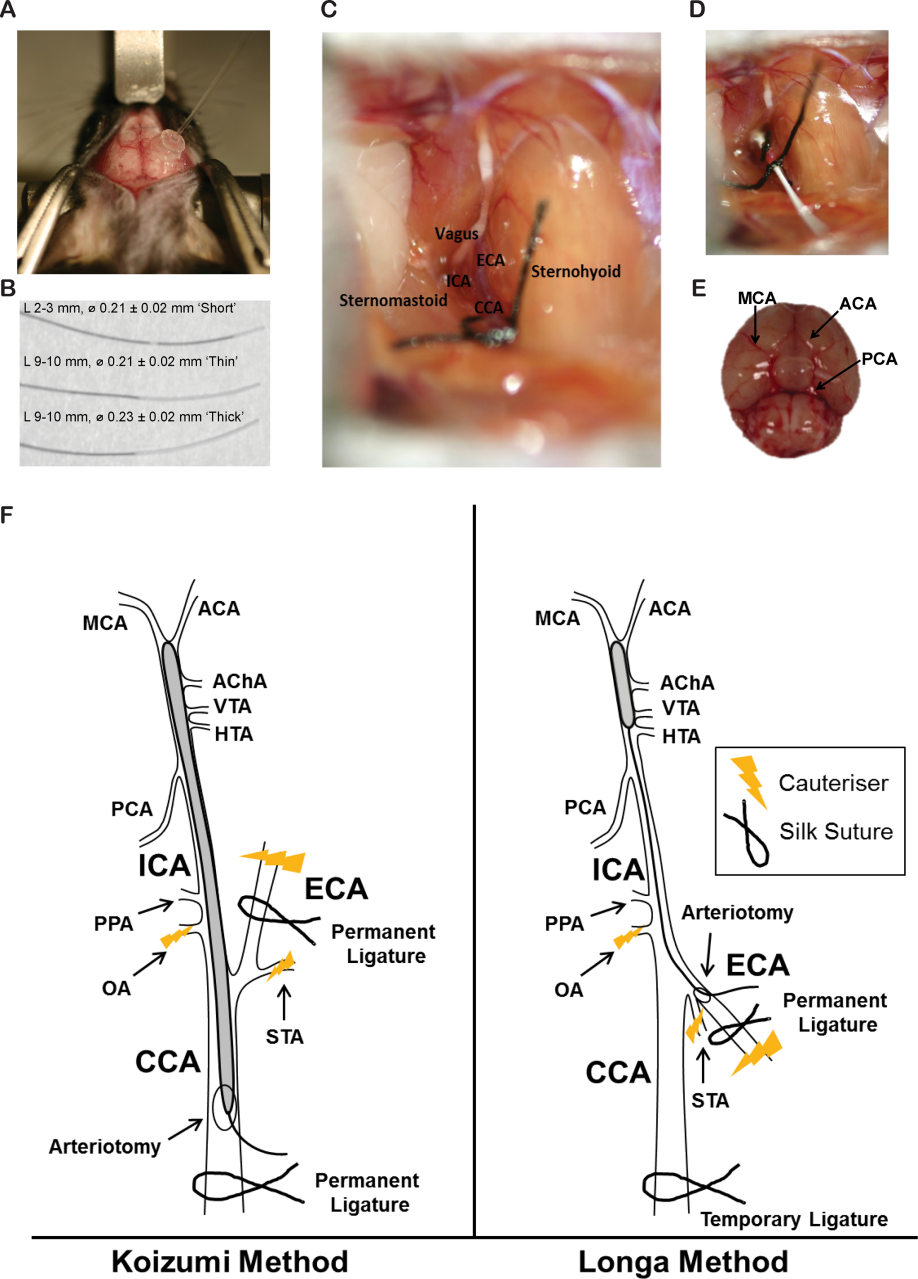
(A) Placement of laser-Doppler probe at Antero-Posterior (AP) ~-1.0 and Medio-Lateral (ML) ~3.0 from bregma, to measure blood flow in the territory supplied by the right MCA. (B) Three different silicone coated filaments, coated for different lengths (L) and with different diameters of coating (⌀), used throughout this study. For simplicity, we have titled these ‘Short’, ‘Thin’ and ‘Thick’. (C) Surgical site during intraluminal filament MCAO identifying the External Carotid Artery (ECA), Internal Carotid Artery (ICA), Central Carotid Artery (CCA). (D) Underside of mouse brain, identifying the Middle Cerebral Artery (MCA), Anterior Carotid Artery (ACA) and Posterior Cerebral Artery (PCA). (F) Koizumi’s method (left) and Longa’s method (right) of the intraluminal filament MCAO (not to scale). Anterior choroidal artery (AChA), HTA, hypothalamic artery (HTA), pterygopalatine artery (PPA), occipital artery (OA), superior thyroid artery (STA), ventral thalamic artery (VTA).

All surgical tools were sterilized for 1 min in a 250°C Dry Glass Bead Sterilizer (Cole-Parmer, #EW-10779-00). The surgical table (a styrofoam platform) and associated equipment were sanitised using 70% (w/v) ethanol. Mice were anaesthetised with ketamine (8.7 mg/ml; Mavlab, Slacks Creek, QLD, http://www.mavlab.com.au) and xylazine (2 mg/ml; Troy Laboratories Pty Ltd, Smithfield, Australia, http://www.troylab.com.au). Both the head and neck of each mouse was shaved then cleaned with an alcohol swab. Mice were placed in a prone position and, using a scalpel blade, a 2 cm midline incision was made on the skin from the nose to between the eyes. Mice were secured, still prone, into a stereotaxic apparatus. The skin was then pulled laterally to expose the skull (Fig 1A). Eye ointment was applied liberally to both eyes.

#### Measurement of local CBF

CBF was monitored before, during and after surgery using a laser-Doppler flowmeter (moorVMS-LDF1, Moor Instruments, Devon, UK). A ‘master’ probe (#VP10M200ST, Moor Instruments, Devon, UK), attached at one end to the laser-Doppler, was attached at the alternate end to a ‘slave’ probe (#P10d, Moor Instruments, Devon, UK). The slave probe was then fed through a silicon electrode holder (#PHDO, Moor Instruments, Devon, UK). Four drops of Loctite Gel (#10762, Loctite) were applied to the silicon holder, just surrounding, but not touching, the slave probe. Optical matching gel (#PMF, Moor Instruments, Devon, UK) was applied to the tip of the slave probe. The silicone holder/slave probe was adhered perpendicular to the right temporal skull at Antero-Posterior (AP) ~-1.0 and Medio-Lateral (ML) ~3.0 from bregma, to measure blood flow in the territory supplied by the right MCA [24] (Fig 1A). Once Loctite Gel firmed the mice, with the laser-Doppler now attached, were carefully released from the stereotaxic apparatus and gently placed supine upon the surgical table, with an area cut from the styrofoam platform to allow room for the attached laser-Doppler probe.

#### MCAO surgery

Mouse body temperature was monitored throughout surgery, occlusion and reperfusion with the use of a rectal thermometer (PhysioSuite®, Kent Scientific Corporation) and maintained between 36.0°C and 38.0°C via the use of a flat reptile heat pad (#HMAT5CN, Reptapets). Refer to S1 Video for a brief video of the surgery. Shaved areas under the chin were sterilised with an alcohol swab and 1 cm midline incisions were made between the manubrium and the jaw, under a stereo dissecting microscope (Leica M50, Leica Microsystems). Underlying submandibular glands were bluntly divided, and retractors (#17000–02, Fine Science Tools) were applied to expose the surgical field beneath the glands. The thin omohyoid muscle sometimes covered the central carotid artery (CCA), and in these cases it was divided to expose the right central CCA. Once identified, the right CCA was separated from the vagus nerve, which lies lateral to it, taking great care not to damage or puncture either structure with surgical tools (Dumont #5/45 #1125–31 and Dumont #JF-5TC Forceps #00632–11, Fine Science Tools). The bifurcation of the CCA into the right external carotid artery (ECA) and right internal carotid artery (ICA) was identified, and the ECA and ICA were isolated from surrounding nerves and fascia (Fig 1C). Damage was minimised to both the trachea and the sternocleidomastoid muscle. Several small nerve fibers are adjacent to the CCA, ECA and ICA, including the main branches of the vagus nerve and hypoglossal nerves and great care was also taken not to cauteriser, pinch, or cut these throughout surgery. The superior thyroid artery (STA) and the Occipital Artery (OA) were both isolated and cauterised (#18010–00, Fine Science Tools, S1 Video).

Next, the ECA was permanently ligated as distal as possible from the bifurcation of the CCA, with silk sutures (Dynek, USP 6/0, #S604), then cauterised. In C57BL/6JAusb a fat pad is often adhered to, and overlays the ICA. This was isolated and either cauterised or clipped away with the use of reverse action scissors (#15000–08, Fine Science Tools), taking care not to damage the ICA. At this point some operators suggest removal of the pterygopalatine artery, which bifurcates from the ICA [8], however due to surgical difficulty reaching it in mice and possible adverse side effects of attempting to do so [19], this was not attempted. From here, the Koizumi and Longa methods diverge.

#### Koizumi method

The CCA was ligated permanently 5 mm from the bifurcation to the ECA and ICA resulting in a reduction in CBF, detected by laser-Doppler. A second loose collar suture was tied around the CCA, 2 mm from the bifurcation. A microvascular clamp (#00325–00, #12018–12, Fine Science Tools) was applied to the ICA. An arteriotomy was performed between the two sutures around the CCA using reverse action scissors. Either one of 3 silicone coated filaments (#60SPREPK5, Doccol (Fig 1B)), were introduced into the arteriotomy and advanced into the right ICA, until they reached the microvascular clamp. The microvascular clamp was then removed to allow insertion of the filament towards the MCA (Fig 1F). Correct placement of the filament at the MCA was confirmed by a sudden drop in CBF, detected by laser-Doppler. The filament was advanced 0.5 mm past the first appearance of the drop to ensure it sat over the origin of the MCA. The loose collar suture was tightened around the inserted filament to prevent movement during the occlusion period. Mice were left supine throughout the occlusion period, to prevent the risk of filament movement, and rectal temperature and CBF were continuously monitored. Following the desired occlusion period (0 min, 15 min, 30 min, 45 min or 60 min), the filament was withdrawn as far as the bifurcation, and the microvascular clamp reapplied to the ICA. The filament was then fully withdrawn and the loose collar suture was tightened around the CCA, to prevent backflow through the arteriotomy. The microvascular clamp was then removed. Sham surgeries were achieved by inserting the filament to the MCA, then immediately withdrawing. Following withdrawal of the filament, CBF was continuously recorded for up to 5 min, to confirm reperfusion. Surgical time was 15–25 min.

#### Longa method

The CCA was ligated temporarily 5 mm from its bifurcation to the ECA and ICA resulting in a drop in CBF, detected by laser-Doppler. A second loose collar suture was tied around the ECA as close to the ICA/ECA bifurcation as possible. A microvascular clamp was applied to the ICA. An arteriotomy was performed between the cauterised stump of the ECA, and the loose collar suture. Either one of the 3 silicone coated filaments (Fig 1B) were introduced into the arteriotomy and advanced up the right ICA, toward the microvascular clamp. The loose collar suture was tightened around the filament and the microvascular clamp was then removed to allow insertion of the filament towards the MCA. As with the Koizumi method, correct placement of the filament at the MCA was confirmed by a sudden drop in CBF, detected by laser-Doppler and the filament was advanced 0.5mm past the first appearance of the drop to ensure it sat over the origin of the MCA. The loose collar suture was then tightened around the inserted filament to prevent movement during the occlusion period. Once the filament was withdrawn following occlusion, the loose collar suture was completely closed around the ECA to prevent backflow through the arteriotomy. The temporary suture around the CCA was then removed, allowing reperfusion through the right CCA. Again, CBF was recorded for 5 min to confirm reperfusion. Surgical time was 20–30 min.

#### Post-surgery

Following surgery the laser-Doppler probe was removed and both neck and head incision sites were sutured. A 0.5 mL saline injection was given subcutaneously to each mouse as fluid replacement immediately post-surgery. Mice were returned to home cages to recover, single-housed and with one half of the cage placed on a heating pad, allowing mice to choose their environment during recovery. Recovery times were 30 min, 4 h, 12 h or 24 h. To assist recovery mice were provided with recovery gel (#72-01-1062, ClearH_2_O) and chow softened in water.

### Calculating Body Weight Loss, CCAO, MCAO and Reperfusion values

**Fig 2:**
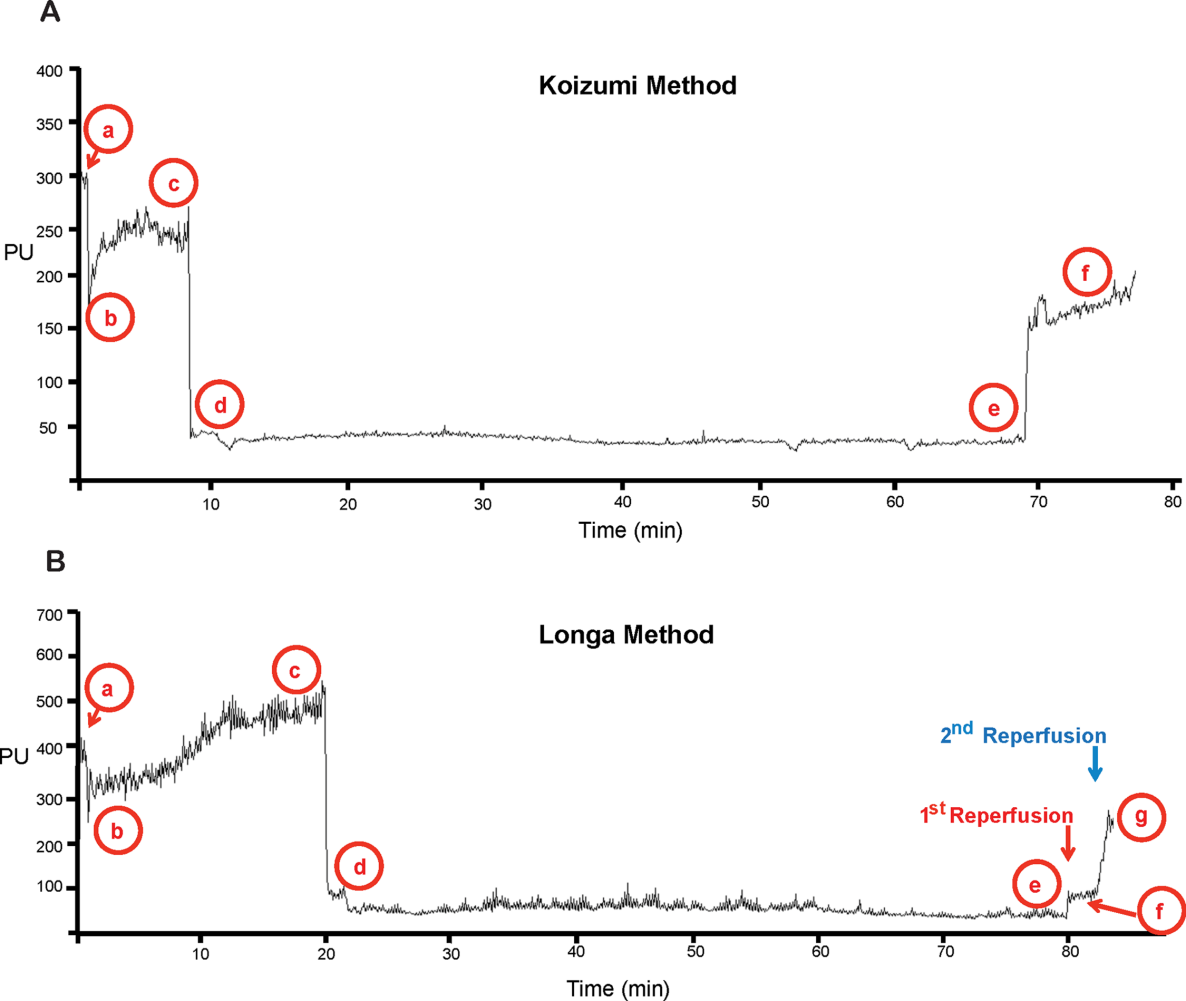
Representative laser-Doppler flowmetry during Koizumi or Longa intraluminal filament MCAO (A) Representative laser-Doppler flowmetry during Koizumi’s method of intraluminal filament middle cerebral artery occlusion (MCAO). (B) Representative laser-Doppler flowmetry during Longa’s method of intraluminal filament MCAO. Perfusion Units (PU) are arbitrary units of cerebral blood flow. a. Baseline. b. Immediately post CCAO. c. Immediately pre-MCAO. d. Immediately post-MCAO. e. Immediately pre-MCA reperfusion. f. 5 min post-MCA reperfusion. g. CCA reperfusion (Longa method only).

Alterations in body weight 4 h, 12 h or 24 h post-reperfusion were expressed as a percentage of baseline body weight, measured immediately prior to surgery. Using continuous laser-Doppler flowmetry the impact of unilateral central carotid artery occlusion (CCAO), MCAO and MCA/CCA reperfusion on cerebral blood flow in the territory supplied by the right MCA, was measured. Arbitrary values (denoted as perfusion units, PU, by the laser-Doppler) for CBF were used to calculate relative changes in CBF immediately pre-CCAO, immediately post-CCAO, immediately pre-MCAO, immediately post-MCAO, immediately pre-MCA reperfusion and 5 min post-MCA reperfusion. Average values were calculated across 30 s intervals, except when measuring immediately post-MCAO, where CBF was calculated across the initial 5 s, as values rise following this period, obscuring the initial drop [25] (Fig 2). There was an additional value, CCA reperfusion, obtained each time the Longa method was employed, measured by averaging 30 s intervals, 5 min following CCA reperfusion.

Alterations in CBF following CCAO and MCAO were expressed as a percentage of baseline CBF, prior to CCAO, reflecting the alteration in CBF in the MCA territory from the initial arbitrary baseline value prior to any manipulation from the CCAO or MCAO. The change in CBF over occlusion was measured as a fold change between the arbitrary values of CBF from immediately post-MCAO to immediately pre-reperfusion. Reperfusion was calculated in two ways; 1. Reperfusion was calculated via a method traditionally used, by determining percentage increase in CBF following reperfusion, relative to the baseline immediately prior to MCAO and 2. Reperfusion was calculated via a modified method, due to the tendency of the arbitrary values of CBF to gradually reduce over occlusion periods, by expressing it as a fold increase in CBF following MCA reperfusion, normalised against the original fold decrease following MCAO and compared to traditionally calculated values. Thus, via this method a reperfusion of 1 represents complete reperfusion back to the level of CBF prior to MCAO. I.e. (see Fig 2A and 2B for explanation of letters a-g);

CCAO(Longa and Koizumi)=b/a*100

MCAO(Longa and Koizumi)=d/a*100

MCAO Reperfusion(Koizumi,‘traditional’calculation)=(f-e)/c-d)

MCAO Reperfusion(Koizumi,‘modified’calculation)=(f/e)/(d/c)

CCAO Reperfusion following MCAO Reperfusion(Longa,via‘traditional’calculation)=(g-e)/c-d)

CCAO Reperfusion following MCAO Reperfusion(Longa,via‘modified’calculation)=(g/e)/(d/c)

Fold change in PU(CBF)over the occlusion period(Longa and Koizumi):d/e

### Neurological Severity Scoring

All neurological severity scoring (NSS) was carried out at 4 h after reperfusion, for animals sacrificed at 4 h, 12 h after reperfusion for animals sacrificed at 12 h, or 24 h after reperfusion for animals sacrificed at 24 h. Animals sacrificed after 30 min reperfusion were not neurologically scored as they were still under anaesthetic and motor movements were not yet restored. Mice were scored as described previously [26] according to a 6 point scale, by an individual blinded to experimental group; 0, normal; 1, mild turning behaviour with or without inconsistent curling when picked up by tail, <50% attempts to curl to the contralateral side; 2, mild consistent curling, >50% attempts to curl to contralateral side; 3, strong and immediate consistent curling, mouse holds curled position for more than 1–2 s, the nose of the mouse almost reaches tail; 4, severe curling progressing into barrelling, loss of walking or righting reflex; 5, comatose or moribund.

### Histological evaluation with 2,3,5-triphenyltetrazolium chloride (TTC)

After 30 min, 4 h, 12 h or 24 h of reperfusion, mice were deeply anaesthetised with 5% isoflurane and euthanised by cervical dislocation. Brains were extracted, placed within a brain matrix (#BSMAA001-1, Zivic Instruments) and ten 1 mm coronal sections were cut. Sections were incubated in 2% TTC in 1 x phosphate buffered saline (PBS) for 10 min at 37°C. After 5 min of incubation sections were flipped to ensure even staining on both sides. Sections were washed once in 1 x PBS, and then fixed in 10% neutral buffered formalin solution at 4°C until imaging.

### Volumetric assessment of ischaemic injury

Of the ten coronal levels cut, only the first eight were quantified, as they contained territories at risk of ischemic injury following MCAO. Brain regions were denoted based on the Comparative Cytoarchitectonic Atlas of the C57BL/6 and 29/Sv Mouse Brains [27] (S1 Fig). An investigator blinded to experimental groups conducted planimetric measurements using ImageJ. Quantification of lesions for each section was made via the indirect Swanson formula, to account for edema, as edema can affect the accuracy of lesion estimation. Volumes were calculated for both the top and bottom of each section. The top and bottom lesion volumes were then averaged to provide an overall lesion volume assessment for each section. The estimated total ipsilateral cortex volume for each brain was then derived from summation of the lesion volumes in each of the each sections, before being expressed as a percentage of the total cortex volume in the control hemispheres ± the standard error of the mean (SEM), using the Swanson formula [28]. The Swanson formula relies upon the left and right brain volumes being identical. To ensure this, the left and right lesion volumes were also analysed in all sham animals. See S1 Fig for details of area A and B.

$$\begin{array}{l} \%\text{infarction}=100\times (\text{volume left hemisphere}\;\left(\text{Area}\;‘\text{A}’\right)-\text{volume non}\;-\;\text{infarcted right}\\ \text{hemisphere}\;\left(\text{Area}\;‘\text{B}’\right)/\;\text{volume left hemisphere}\;\left(\text{Area}\;‘\text{A}’\right) \end{array}$$

### Statistical analysis

Statistical analysis was performed using GraphPad Prism Version 6.0 (GraphPad Software, Inc), SPSS (Graduate pack) (SPSS Inc., Chicago, IL, http://www.spss.com), or STATA (STATACorp 2015). For Neurological severity, scores were square root transformed to produce a normal distribution. Outliers were detected in ipsilateral lesion data via Grubbs’ test and removed accordingly. Differences between means were assessed, as appropriate, by, one-, two-or three-way ANOVA, followed by Bonferroni *post-hoc* analysis. For analysis of weight loss, three-way ANCOVA was performed with starting weight as a covariate. Correlations were assessed by simple linear regression.

## Results

### Filament width and time point of collection post-reperfusion do not alter the volume of ischemic lesions

Previous studies have shown that the volume of ischemic lesion grows between 0–24 h, peaking at 24 h, when assessed by TTC staining following the intraluminal filament model of MCAO in mice [14]. However, contrary results have suggested the volume of ischemic lesions following a permanent MCAO in rats is not significantly different when assessed 30 min after reperfusion, compared with 24 h [29]. We therefore tested whether the collection time after reperfusion impacts the volume of resulting ischemic lesions in the acute phase of recovery (0–24 h). Mice underwent a 60 min intraluminal filament MCAO using the Koizumi method, or sham surgery. 60 min occlusions were initially chosen based on preliminary results indicating high mortality following longer periods of occlusion (S1 Table) and was in line with commonly used occlusion periods in other studies in mice [2]. Following occlusion, mice were euthanised and analysed via TTC staining at various time points after reperfusion (30 min, 4 h, 12 h and 24 h).

Previous studies have also suggested the tip width of intraluminal filaments used for MCAO can alter resulting volumes of ischemic lesions [30]. Recently, two different tip widths for occluding filaments have been utilised in mice, 0.21mm [31–33] and 0.23mm [8], although the type of filaments used in both rats and mice have not only varied in size and composition, but also tensile strength, elasticity, material and length of coating [34]. We therefore also performed the intraluminal filament MCAOs using standardised ‘thick’ (0.23 mm) and ‘thin’ (0.21 mm) silicone coated monofilaments, produced by the Doccol corporation, coated with silicone for 9–10 mm (Fig 1B).

**Fig 3:**
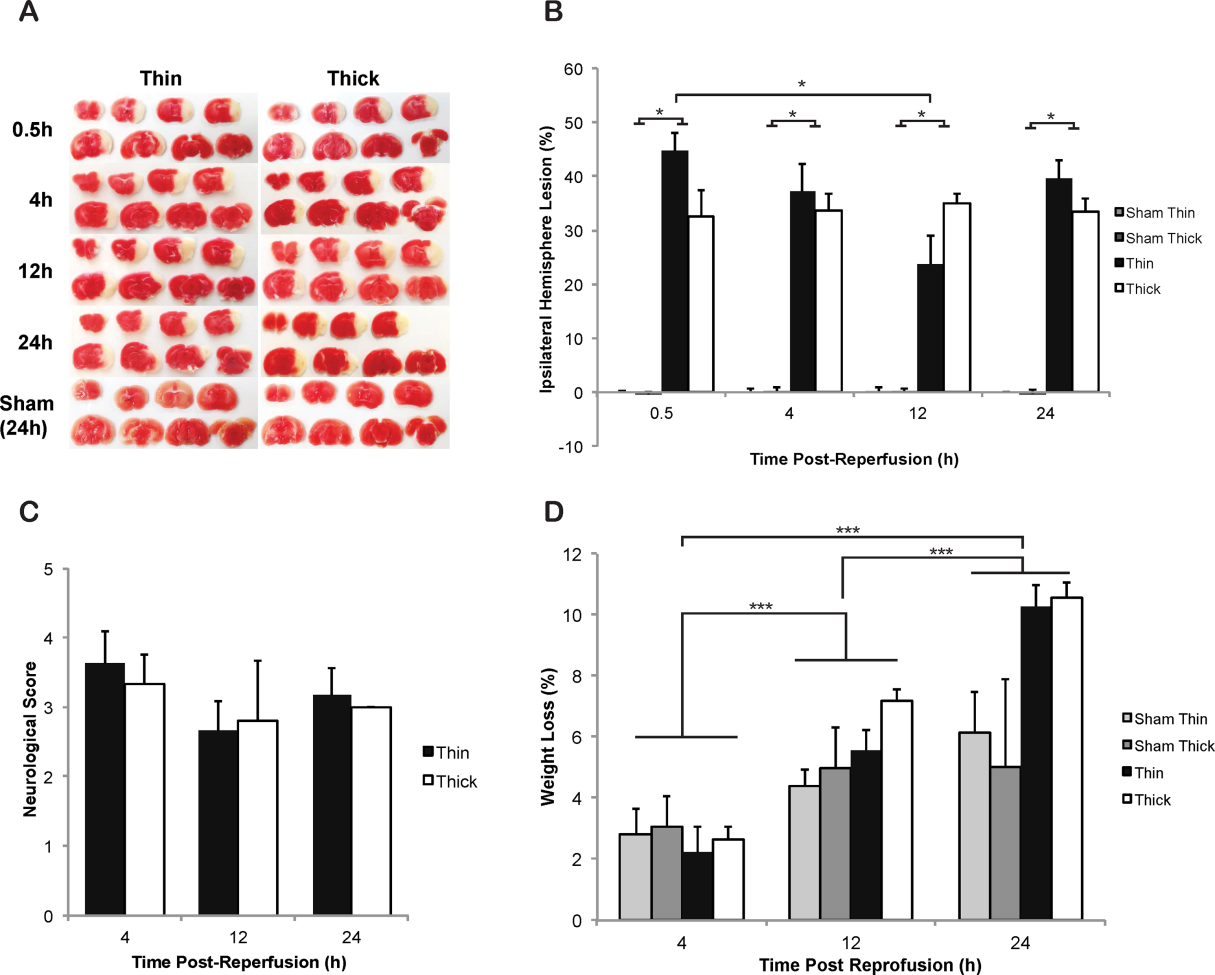
Post-reperfusion time-course of ischemic lesion volume detected by TTC following 60 min intraluminal filament MCAO using the Koizumi method (A) Representative TTC stained brain sections indicating areas of healthy tissue (red) and ischemic injury (white) for each group. (B) Total volume of ischemic lesion in the ipsilateral hemisphere, expressed as a percentage of the total contralateral hemisphere volume, 30 min, 4 h, 12 h and 24 h after reperfusion following sham surgeries or 60 min MCAOs, with thin or thick silicone coated filaments, via the Koizumi method. (C) No alteration in neurological severity scores between animals assessed at 4 h, 12 h and 24 h after reperfusion, following a 60 min occlusion. (D) Body weight loss post-MCAO significantly increased from 24 h as compared to 4 h and 12 h after reperfusion. Each value represents the mean ± the standard error of the mean (SEM). *p < 0.*05*. N = 3–4 for sham surgeries and n = 5–8 for animals undergoing occlusion.

There was no significant difference between the total volume of left and right cerebral hemispheres in animals that underwent sham surgery, a critical factor when employing the Swanson correction for ischemic injury analysis. There was also no significant difference in age or starting weight between any groups undergoing occlusion (S8, S9 and S10 Tables). A 60 min occlusion produced a significant lesion compared with sham mice across all times after reperfusion (Fig 3A and 3B, *p < 0*.*05*). The total percentage of ischemic lesions in the ipsilateral hemisphere was not significantly different at any time point assessed after reperfusion, when comparing animals undergoing occlusion with thin or thick filaments, indicating 0.21 and 0.23 mm diameter filaments are both capable of inducing large, reproducible ischemic lesions (Fig 3A and 3B). However, there was a significantly larger lesion volume in animals assessed at 30 min after reperfusion (44.6% ± 3.4%) than animals assessed at 12 h after reperfusion (23.8% ± 5.3%), after undergoing 60 min occlusion with thin filaments (Fig 3B, *p* < 0.05). Lesion volume was largest by percentage of the total ipsilateral hemisphere in coronal level 3 (corresponding to Bregma 0.0), compared with the other 7 coronal levels, in the thin 4 h, thin 12 h, thin 24 h and thick 4 h groups. Lesion volume was largest in coronal level 4 (Bregma -1.0) in the thick 30 min, thick 12 h and thick 24 h groups, while the thin 30 min group had the largest area of lesion in coronal level 5 (Bregma -2.0) (S2A Fig). This indicates the core infarct was most often between Bregma 0.0 and -1.0, regardless of time after reperfusion.

To determine the effect of filament width and time after reperfusion, following MCAO, on ischemia in various brain structures, we assessed the total number of animals with lesions in areas of the brain commonly injured during intraluminal MCAO in rodents [2]. Ischemic lesions were observed in the striatum and cortex in all animals undergoing 60 min occlusion with either a thin or thick filament and at every time point after reperfusion (S7 Table). 8/14 and 6/15 (thin and thick filament occlusions combined) animals assessed at 30 min and 4 h after reperfusion, respectively, had ischemic lesions in the dorsal hippocampus and 8/14 and 8/15 had evidence of ischemic lesions in the ventral hippocampus at 30 min and 4 h after reperfusion respectively. 7/14 and 6/13 had lesions in the thalamus at 30 min and 4 h after reperfusion respectively. However, fewer animals had injury in these areas at 12 h and 24 h after reperfusion (2/11 in dorsal and ventral hippocampus, and 3/11 and 2/11 in the thalamus for 12 h and 24 h respectively). On the contrary, more animals assessed at 12 h and 24 h after reperfusion had ischemic lesions in the amygdala in comparison to the 30 min and 4 h groups (7/11 and 11/11 vs. 4/14 and 1/15), suggesting injury in this area may appear later in the evolution of the ischemic infarct.

Following intraluminal MCAO, animals present with neurological deficits that can be assessed on a simple 6 point scale [26]. In our study there were no significant differences in neurological scores between animals occluded with thin and thick filaments, or between any animals assessed at different time points after reperfusion (Fig 3C). No sham animals showed any sign of lesion or neurological deficit. Weight loss was significantly different between animals euthanised at 24 h after reperfusion, and animals euthanised at 4 h or 12 h after reperfusion (Fig 3D, *p < 0*.*05*), but there was no difference in weight loss between thin and thick groups. There were also no significant differences in relative levels of CBF following CCAO, MCAO or reperfusion (based on the modified calculation) in any groups, indicating a standard consistency in surgical technique across all groups (Table 1). There was also no significant difference in reperfusion based on the traditional calculation between any groups undergoing occlusion. However reperfusion was significantly higher in sham groups compared to occlusion groups (F_(15,59)_ = 9.090, *p < 0*.*05*), based on the traditional method, indicating that if the drop in CBF over occlusion (also evident in Table 1) is not controlled for in the calculation, as it is in the modified method, reperfusion appears significantly decreased.

**Table 1:** Relative alterations in CBF following CCAO, MCAO, during occlusion and following reperfusion, for thin and thick filaments at 30 min, 4 h, 12 h, and 24 h after reperfusion.

Recovery Time (h)	Filament	n	CCA	MCAO	CBF Reduction over occlusion (Fold Change)	Reperfusion (modified calculation)	Reperfusion (traditional calculation)
0.5	Sham Thin	3	30.41±2.9	76.49±4.47	1	0.949±0.19	92.19±3.81*
	Sham Thick	3	29.13±10.28	77.82±2.33	1	1.01±0.01	101.75±2.01*
	Thin	7	29.61±2.41	78.42±1.8	3.87±1.15	1.58 ± 0.57	38.35±6.62
	Thick	7	33.36±5.35	75.48±3.72	2.73±0.87	1.1 ± 0.25	42.69±5.94
4	Sham Thin	3	44.48±10.88	81.82±5.03	1	1.07±0.02	108.16±2.40*
	Sham Thick	3	39.8±1.49	81.82±5.03	1	1.04±0.04	105.50±6.33*
	Thin	8	26.16±4.74	82±1.66	1.72±0.24	0.87±0.13	55.41±14.45
	Thick	7	22.78±3.67	74.43±3.4	1.56±0.17	0.64±0.09	31.53±7.31
12	Sham Thin	3	32±2.38	77.82±1.16	1	1.07±1.063	109.28±0.14*
	Sham Thick	3	41.97±3.36	86.85±2.04	1	1.013±0.01	113.27±5.62*
	Thin	6	23.23±0.57	79.09±3.7	1.81±0.31	0.9±0.17	48.25±10.42
	Thick	5	25.93±6.5	68.12±3.04	2.04±0.35	0.8±0.09	35.85±3.99
24	Sham Thin	3	34.14±2.2	81.179±3.09	1	1.03±0.02	104.50±2.58*
	Sham Thick	3	45.28±7.92	77.92±4.34	1	1.02±0.02	101.68±2.59*
	Thin	6	28.54±3.9	72.56±6.94	1.58±0.23	0.81±0.15	49.68±13.94
	Thick	5	32.23±3.39	80.61±3.63	2.4±0.45	0.90±0.06	40.68±6.83

Each value represents the mean ± the standard error of the mean (SEM)

* *p < 0*.*05*.

Mortality is known to be high during the acute phase of recovery (0–24 h) from intraluminal MCAO in mice [35]. Consistent with this, our data revealed the percentage of animals surviving decreased between 0–24 h. There was 87.5% and 100% survival at 30 min, 75% and 70% survival at 4 h, 60% and 83.3% survival at 12 h and only 37.5% and 26.3% survival at 24 h after reperfusion, for animals occluded with thin and thick filaments, respectively (S4 Table). Considering the high survival rate in all animals undergoing sham surgeries in this study (47/49 (96%)), death was considered to result from ischemic injury, rather than surgical complications (S4, S5 and S6 Tables).

### Ischemic lesion volume increases with extended occlusion periods

It is important to establish how lesion volume evolves following different length MCAOs in mice, due to widespread variability between studies. Considering lesion volumes in pre-clinical MCAO work are used to compare to the volume of injury following MCAO in humans, it is also important to determine which occlusion time in rodents can reproduce similar size lesions to those seen in human MCAO. Mice therefore underwent varying levels of MCAO (0 min, 15 min, 30 min, 45 min and 60 min) with ‘thick’ intraluminal filaments, were euthanised and ischemic lesion volumes were analysed via TTC staining at 4 h and 24 h after reperfusion.

**Fig 4:**
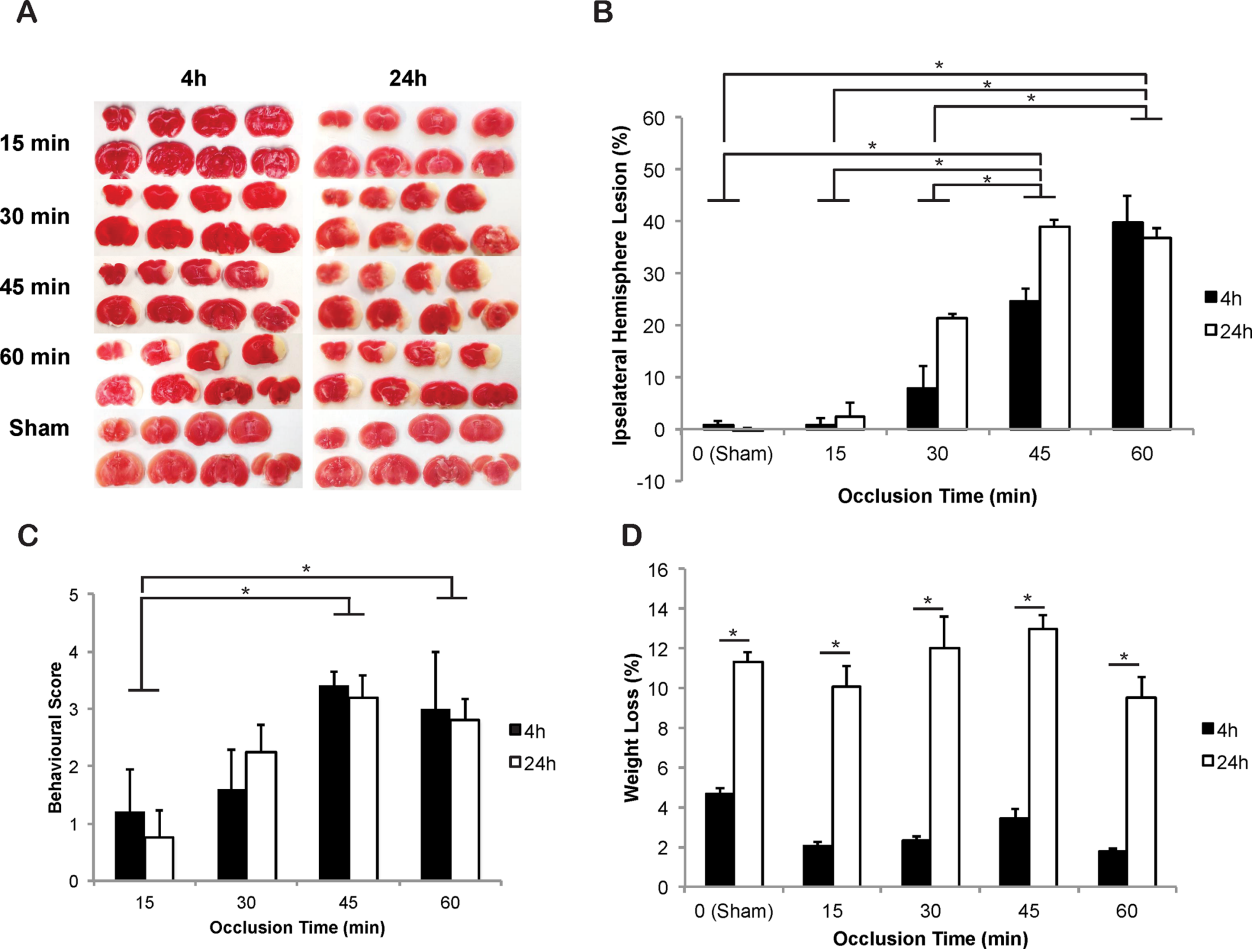
Ischemic lesion volume detected by TTC following 15 min, 30 min, 45 min or 60 min intraluminal filament MCAO using the Koizumi method (A) Representative TTC stained brain sections indicating areas of healthy tissue (red) and ischemic injury (white) for each group. (B) Total volume of ischemic lesions in the ipsilateral hemisphere, expressed as a percentage of the total contralateral hemisphere volume, measured at 4 and 24 h after reperfusion, following 0 min, 15 min, 30 min, 45 min or 60 min of MCAO with a thick silicone coated filament, via the Koizumi method. (C) Significant increases in neurological severity scores following 45 or 60 min MCAO, compared to 15 min MCAO. (D) Body weight loss significantly increased from 4 h to 24 h after reperfusion in every group. Each value represents the mean ± the standard error of the mean (SEM). *p < 0.*05*. N = 3 for sham surgeries and n = 4–7 for animals undergoing occlusion.

A two-way ANOVA revealed no significant interaction of post-reperfusion and occlusion time (F_(4,37)_ = 2.277, *p > 0*.*05*), therefore the main effects were assessed. We revealed no effect of time after reperfusion on lesion size (F_(1,37)_ = 3.630, *p > 0*.*05*), however there was a significant difference in occlusion time (F_(4,37)_ = 3.630, *p < 0*.*05*), indicating the time of occlusion alters lesion size outcome. Post-hoc analysis with Bonferroni corrections indicated significant differences between the total percentage of ischemic lesion volume in the ipsilateral hemisphere following 45 min occlusion after 4 h and 24 h reperfusion (24.6% ± 2.5% and 39.1% ± 1.25%, respectively), compared with sham (0 min), 15 min and 30 min occlusions after 4 h and 24 h reperfusion (0.7% ± 0.8% and -0.2% ± 0.5%, 0.9% ± 1.2% and 2.3% ± 2.7%, 7.7% ± 4.4% and 21.4% ± 0.79%, respectively, Fig 4A and 4B, *p* < 0.05) 7.7% ± 4.4%, Fig 4A and 4B, *p* < 0.05). The same was true for 60 min occlusions at 4 h and 24 h (39.7% ± 5.4% and 36.8% ± 2.0%, respectively) compared to 0, 15 and 30 min occlusions (*p* < 0.05). However there was no significant difference in total ischemic lesion volume after 4 h or 24 h when comparing 45 and 60 min occlusions (Fig 4B). Lesion volume was largest by percentage of the total ipsilateral hemisphere in coronal level 3 (corresponding to Bregma 0.0), compared with the other 7 coronal levels in the 15 min occlusion, 24 h after reperfusion, 30 min occlusion, 4 h and 24 h after reperfusion and the 45 min, 4 h after reperfusion groups (S2B Fig). Coronal level 4 (Bregma -1.0) had the largest percentage lesion for the remaining groups. The core infarct was therefore consistently between Bregma 0.0 and -1.0, independent of occlusion length, or time after reperfusion.

As above, we assessed the effect of occlusion on ischemic lesions in various brain structures commonly affected by MCAO in rodents. Following 15 min occlusion, only 3/5 and 0/5 had ischemic lesions in the cortex, and 2/4 and 1/4 mice had ischemic lesions in the striatum, 4 h and 24 h after reperfusion, respectively. After 30 min occlusion and 4 h after reperfusion 3/5 and 2/5 mice had ischemic lesions in the cortex and striatum, respectively. Ischemic lesions were observed in the striatum and cortex in 100% of other animals, regardless of occlusion or time after reperfusion (S7 Table). There was no ischemic injury noticeable in any other brain region in mice undergoing 15 min and 30 min occlusions, regardless of time after reperfusion. The dorsal and ventral hippocampus were injured in 1/5 and 4/5 mice subjected to 45 min of occlusion, 4 h and 24 h after reperfusion respectively whereas the dorsal and ventral hippocampus were injured in 3/5 and 1/5 mice undergoing 60 min occlusion 4 h and 24 h after reperfusion, respectively. Only mice undergoing 45 min or 60 min of occlusion showed any evidence of ischemic lesions in the thalamus, although thalamic injury was more prevalent following a 60 min stroke, after 4 h reperfusion (3/5 animals) than all other groups. Also, only 45 min occlusion with 24 h reperfusion (5/5 mice), and 60 min occlusion with 4 h (4/5 mice) or 24 h (4/5 mice) reperfusion, produced any injury in the amygdala. This indicates that longer occlusion times increase the likelihood of ischemic injury in the thalamus, hippocampus and amygdala, although lesions in the amygdala are more noticeable after 24 h of reperfusion, rather than 4 h, supporting the trend observed in the post-reperfusion time-course above. However, in contradiction to the results obtained 24 h after a 60 min occlusion, hippocampal lesions were still visible after 24 h reperfusion in the majority of animals undergoing a 45 min stroke (4/5), suggesting the prevalence of ischemic injury in the hippocampus may not always be reduced after 24 h reperfusion.

The neurological scores following the varying occlusion periods largely followed a similar pattern to the lesion data, with significant differences between both 45 min and 60 min occlusions when compared with 15 min occlusions (Fig 4C, *p < 0*.*05*). No sham animals showed signs of lesion or neurological deficit. Weight loss was significantly different between animals euthanised at 24 h after reperfusion, and those euthanised at 4 h after reperfusion for every occlusion time (Fig 4D, *p* < 0.05). There were no significant differences in relative levels of CBF following CCAO, MCAO or reperfusion (based on the modified calculation) in any groups, once again indicating a standard consistency in surgical technique across all groups (Table 2). There was also no significant difference in reperfusion based on the traditional calculation between any groups undergoing occlusion, although there was a trend toward lower reperfusion the longer the occlusion time (F _(9,36)_ = 3.81, *p* = 0.0803).

**Table 2:** Relative alterations in CBF following CCAO, MCAO, during occlusion and following reperfusion for 15 min, 30 min, 45 min and 60 min occlusions measured at 4 h or 24 h after reperfusion.

Occlusion Time (min)	Recovery Time (h)	n	CCA	MCAO	CBF Reduction over occlusion (Fold Change)	Reperfusion (modified calculation)	Reperfusion (traditional calculation)
0	4	3	45.2±2.24	86.67±2.04	1	1.11±0.02	113.1±2.68
	24	3	28.27±6	79.31±1.76	1	1.04±0.03	105.1±4.50
15	4	5	32.56±3.31	79.66±2.2	0.88±0.09	0.69±0.08	68.4±11.11
	24	4	29.68±1.93	79.11±2.47	0.94±0.07	0.78±0.11	79.5±18.55
30	4	5	25.48±1.93	78.42±3.23	1.73±0.22	1.37±0.40	87.2±23.18
	24	4	29.68±4.77	82.86±2.48	1.26±0.07	1.03±0.15	82.7±12.45
45	4	5	23.52±5.93	74.54±1.91	2.8±0.71*	1.68±0.48	66.7±15.67
	24	5	30.35±3.61	74.23±2.43	2.11±0.4	1.09±0.22	56.7±18.77
60	4	7	22.05±3.77	79.76±3.82	2.34±0.43	0.85±0.08	40.5±6.3
	24	5	25.60±2.86	69.14±7.16	1.16±0.13	1.1±0.24	88.3±19.7

Each value represents the mean ± the standard error of the mean (SEM).

*p < 0.*05*

There was a significant difference in the decrease of CBF over time in the 45 min occlusion group, with a 4 h reperfusion, compared to both 15 min groups (Table 2, F _(9,36)_ = 3.542 p *<* 0.01), with a trend towards a significant increase in the 45 min occlusion group, with a 24 h reperfusion, mirroring the trend seen with reperfusion calculated via the traditional method. Once again, this indicates the drop in CBF over occlusion time appears to heavily influence the level of reperfusion based on the traditional calculation. There was also a trend toward increased CBF reduction over time in the 30 min and 60 min occlusion groups compared to the 0 min (sham) and 15 min occlusions, indicating the longer the occlusion the more likely a reduction in CBF.

The percentage of animals surviving occlusion decreased as occlusion time increased. There was 83.3% and 100% survival after 15 min occlusion, 4 h and 24 h reperfusion, 83.3% and 41.7% after 30 min occlusion, 4 h and 24 h after reperfusion, 85.7% and 50% after 45 min occlusion, 4 h and 24 h after reperfusion and 81.8% and 38.5% after 60 min occlusion, 4 h and 24 h after reperfusion, respectively (S5 Table).

### The Longa method of intraluminal filament MCAO produces more complete reperfusion than the Koizumi method without altering ischemic infarct size at 4 h reperfusion

As mentioned earlier, there are two key differences between the original intraluminal filament methods of Koizumi and Longa; 1. The entry point of the intraluminal filament, which is via the CCA in Koizumi, but the ECA in Longa, allowing reperfusion via both CCAs in Longa’s method and 2. The type of intraluminal filament employed in the Koizumi method was initially a consistent width 5 mm silicone coated filament, differing to that of the Longa method which utilised a heat blunted suture with an enlarged width only at the tip [36]. Despite the use of both methods throughout the literature, in both rat and mouse studies, it is surprising that very few studies exist comparing the two methods side-by-side.

In this study we therefore compared the Longa and Koizumi methods of intraluminal MCAO, and aimed to determine if coating length of the intraluminal filament, or the difference in entry point of the filament (ECA vs. CCA) were responsible for any difference in resulting lesion volumes or levels of reperfusion. Animals underwent 60 min MCAO via either the Koizumi, or the Longa method, using either ‘thick’ (silicone coated for 9–10 mm) filaments, similar to those originally used by Koizumi et al., [3] or ‘short’ (silicone coated for 2–3 mm) filaments, coated for a shorter length than those used by Koizumi et al., but more similar to the bulbous-head, heat-blunted filaments used by Longa et al., [4]. Mice were compared at 4 h after reperfusion, as survival was high at this point, reducing the possibility that any potential differences in survival would alter resulting lesion comparisons. Furthermore, our earlier results with the Koizumi method suggested no difference in lesion volume between 4 h and 24 h when assessed by TTC following a 60 min occlusion.

**Fig 5:**
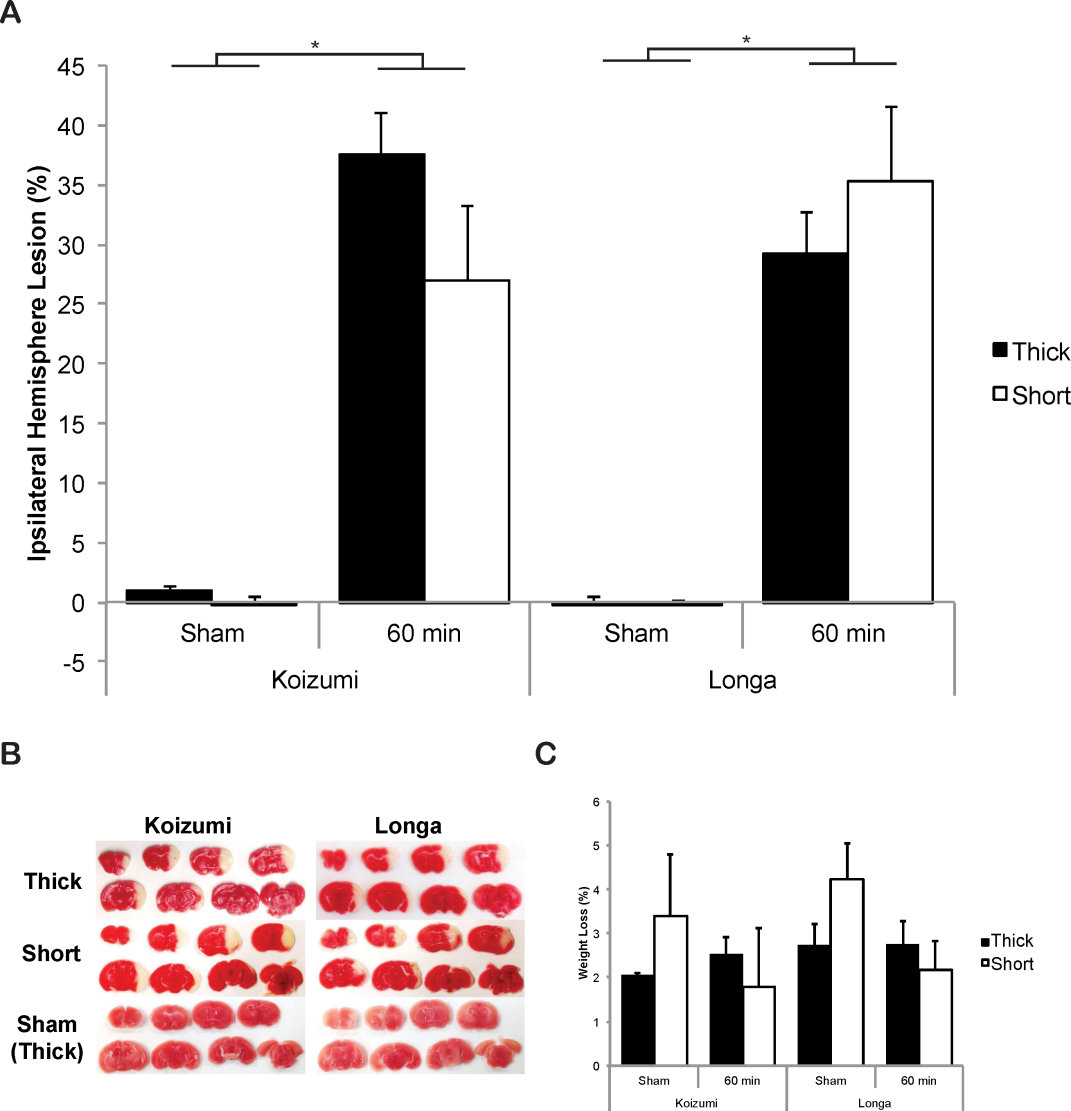
Ischemic injury detected after 4 h reperfusion by TTC following 60 min intraluminal filament MCAO via the Koizumi or Longa method (A) Total volume of ischemic lesion in the ipsilateral hemisphere, expressed as a percentage of the total contralateral hemisphere volume, measured at 4 h after reperfusion, following 60 min MCAO via the Koizumi or Longa method with a thick or short silicone coated filament. (B) Representative TTC stained brain sections indicating areas of healthy tissue (red) and ischemic injury (white) for each group. (C) Body weight loss following either Koizumi or Longa surgeries measured at 4 h after reperfusion. Each value represents the mean ± the standard error of the mean (SEM). N = 3 for sham surgeries and n = 5–6 for animals undergoing occlusion.

There was a similar level of survival following both Koizumi and Longa methods, with both filament types (62.5–83.3%, S6 Table). The total percentage of ischemic lesion volume in the ipsilateral hemisphere was not significantly different between the Longa or Koizumi method, or when using thick or short coated filaments in either model (Fig 5A and 5B). Lesion volumes were largest in coronal levels 3 (Bregma 0.0) for the thick Longa group and coronal level 4 (Bregma -1.0) for all other groups, indicating the core infarct was once again between Bregma 0.0 and -1.0 (S2C Fig).

We compared the various brain regions injured following occlusion in the Koizumi and Longa methods. Ischemic lesions were observed in the striatum and cortex in all animals (S7 Table). There was little difference in the proportion of animals with evidence of injury in the ventral and dorsal hippocampus, thalamus and amygdala across the groups, indicating neither the type of surgery, nor the type of filament altered the areas of injury after 4 h reperfusion.

There were no significant differences in relative levels of CBF following CCAO, MCAO or reperfusion in any Koizumi groups, once again indicating consistency (Table 3). There was however significantly increased reperfusion (via the modified calculation) in animals undergoing the Longa surgeries with short filaments, following the release of the temporary suture occluding the CCA, when compared with reperfusion following the Koizumi method with short filaments (Table 3, *F*_(7,25)_ = 2.639, *p < 0*.*05*), which leaves the right CCA permanently blocked (Table 3). Significantly increased reperfusion in the Longa vs. the Koizumi method was also verified by the traditional calculation of reperfusion, with a significant increase detected when comparing any of the Longa groups to the Koizumi thick filament group (F _(7,25)_ = 3.81, *p* < 0.01).

**Table 3:** Relative alterations in CBF following CCAO, MCAO, during occlusion and following reperfusion for Koizumi vs. Longa at 4 h after reperfusion.

Surgical Method	Filament	n	CCA	MCAO	CBF Reduction over occlusion (Fold Change)	Reperfusion (MCA for Koizumi, CCA for Longa)	Reperfusion (modified calculation)
Koizumi	Sham Thick	3	47.61±4.52	82.3±3.05	1	0.92±0.04	89.8±5.5
	Sham Short	3	25.49±5.50	85.03±2.06	1	1.01±0.01	102.0±1.35
	Thick	6	18.77±3.06	76.54±3	2.53±0.76	0.85±0.17	41.9±10.3
	Short	5	21.28±4.2	74.86±2.93	1.74±0.27	0.86±0.11	53.7±12.9
Longa	Sham Thick	3	19.03±14.59	76.71±4.7	1	1.124±0.12	114.5±14.1*
	Sham Short	3	33±4.32	74.9±5.56	1	1.14±0.07	116.9±8.79*
	Thick	5	24.41±3.13	74.83±2.28	1.66±0.31	1.43±0.14	106.0±18.5*
	Short	5	35.23±4.55	84.79±3.56	1.62±0.21	1.61±0.31*	110.6±16.9*

Each value represents the mean ± the standard error of the mean (SEM).

*p < 0.*05*

### Relative changes in CBF following CCAO, MCAO and reperfusion do not correlate to ischemic infarct volume

In intraluminal filament MCAO studies there is a tendency to remove animals with laser-Doppler calculated MCAO reductions lower than ‘70%’ relative to baseline CBF following MCAO [37–39]. However, to the best of our knowledge there is no study in mice indicating animals with relative alterations in CBF of <70%, calculated using laser-Doppler derived measurements, have different size ischemic lesions to animals with relative alterations in CBF of >70% in the MCAO territory. Determining if this 70% cut-off point is statistically relevant is important as many studies may be unnecessarily removing data on the basis of an arbitrary cut-off point. We therefore analysed whether there was any statistical evidence to support the removal of data on the basis of relative CCAO, MCAO and reperfusion values calculated from laser-Doppler readouts, by correlating these values to resulting lesion volumes in each mouse, via linear regression. The laser-Doppler data was pooled where appropriate, in all animals undergoing the same conditions (i.e. Koizumi surgeries with thick filaments, 60 min occlusion and 4 h recovery, and Koizumi surgeries with thick filaments, 60 min occlusion and 24 h recovery), but where conditions differed, such as the variety of different occlusion periods and recovery times, which can influence the resulting lesions volumes, data was correlated separately.

In all animals included in the study there was a noticeable drop in CBF following CCAO, ranging from 3.5–64% (for all groups the average drop was 29.3% ± 0.9%). Following CCAO, CBF generally increased rapidly back to the level of pre-CCAO CBF, possibly due to immediate reperfusion via the un-occluded left CCA. The level of MCAO ranged from 40–95% (for all groups the average drop was 77.9% ± 0.7%). Although we found the great majority of animals experienced a reduction in CBF of >70% from baseline following intraluminal filament MCAO (138/153 (87.9%)), there were a small proportion of animals with MCAO drops falling between 60–70% (15/153 (9.6%)) and an even smaller proportion with values falling between 40–60% (4/153 (2.5%)).

There was no correlation between the size of CCAO and resulting lesion volume in each group assessed (Table 4). For all groups there was also no significant correlation between the levels of MCAO and reperfusion (calculated via both methods) compared to resulting lesion volume. This indicates that the relative alteration in CBF following MCAO, calculated using laser-Doppler, does not determine the lesion volume. These results therefore provide support for the argument it is unnecessary to remove data on the basis of MCAO drops calculated from laser-Doppler readouts and indicates that the level of reperfusion does not provide any statistical indication of resulting lesion volume.

**Table 4:** Correlations of relative alterations in CBF following CCAO, MCAO, reperfusion, neurological score, weight loss, age and starting weight to ischemic infarct volume.

Occlusion Time (min)	Recovery Time (h)	n	Filament	Surgical Method	CCAO	MCAO	Reperfusion (modified calculation)	Reperfusion (traditional calculation)	Neurological Score	Weight Loss (% of starting weight)	Starting Body Weight	Starting Age
60	0.5	7	Thin	Koizumi	0.1268	0.7003	-0.3991	-0.6205	N/A	N/A	0.2233	0.09996
60	4	8	Thin	Koizumi	0.09871	-0.375	-0.4095	-0.4299	0.1839	0.3474	0.2558	-0.07882
60	12	6	Thin	Koizumi	0.2873	0.5682	0.09023	0.05019	-0.7407	0.7662	0.4453	0.01676
60	24	6	Thin	Koizumi	0.2076	0.6478	-0.3418	-0.4294	0.4349	-0.4070	-0.1856	-0.1225
60	0.5	7	Thick	Koizumi	-0.222	-0.04689	-0.49	-0.518	N/A	N/A	0.04971	0.2553
60	4	20	Thick	Koizumi	0.2835	0.3207	0.3383	0.1962	0.1111	0.06063	0.4602*	0.4344
60	12	5	Thick	Koizumi	-0.0462	0.3353	-0.06473	0.7795	0.8748	-0.7795	0.1223	0.3851
60	24	10	Thick	Koizumi	0.4724	-0.07115	0.0807	0.07662	0.3416	-0.1274	0.07993	0.2628
15	4	5	Thick	Koizumi	0.554	-0.4102	-0.3433	-0.7058	0.1576	-0.03727	-0.1738	0.2323
30	4	5	Thick	Koizumi	-0.4309	0.8331	-0.608	-0.7601	0.7643	-0.7601	-0.1565	-0.8328
45	4	5	Thick	Koizumi	-0.3467	0.3023	-0.03974	-0.7842	0.3102	-0.1438	-0.193	0.7155
15	24	4	Thick	Koizumi	-0.1864	0.2054	-0.7422	-0.8587	-0.7998	0.2103	-0.947	0.3452
30	24	4	Thick	Koizumi	0.773	0.7218	0.0176	-0.321	-0.2203	0.01763	-0.4964	-0.249
45	24	5	Thick	Koizumi	-0.1106	0.7955	-0.5429	-0.5452	-0.3235	-0.3235	-0.6357	-0.7553
60	24	5	Short	Koizumi	-0.5642	-0.8274	0.3913	0.3697	N/A	0.2984	0.4118	-0.1326
60	4	5	Short	Longa	-0.6358	0.1284	-0.8624	-0.4658	-0.8624	-0.4658	-0.5046	-0.8457
60	4	5	Thick	Longa	0.2314	-0.2747	-0.4232	0.2166	N/A	0.5530	-0.28	-0.4075

Values represent r values of correlations.

*p < 0.05

Previous studies of intraluminal filament MCAO have also indicated that the percentage body weight loss post-MCAO [40] can correlate to lesion volume. Starting age and starting body weight may also influence lesion volume, based on the premise that different weight mice have different size arteries, and therefore occluding filaments may be either too big or too small depending on the initial size of a mouse. To determine the influence of these factors in our model we also correlated them final lesion volume. There was no correlation between the neurological score, percentage body weight loss, or starting age and lesion volume in any of the groups assessed. In 16 out of the 17 experimental groups there was also no correlation between starting body weight and lesion volume. However heavier animals had a statistically significant increase in lesion volume following a 60 min occlusion via the koizumi method with thick filaments, assessed 4 h post-reperfusion (r = 0.4602, *p < 0*.*05*).

**Fig 6:**
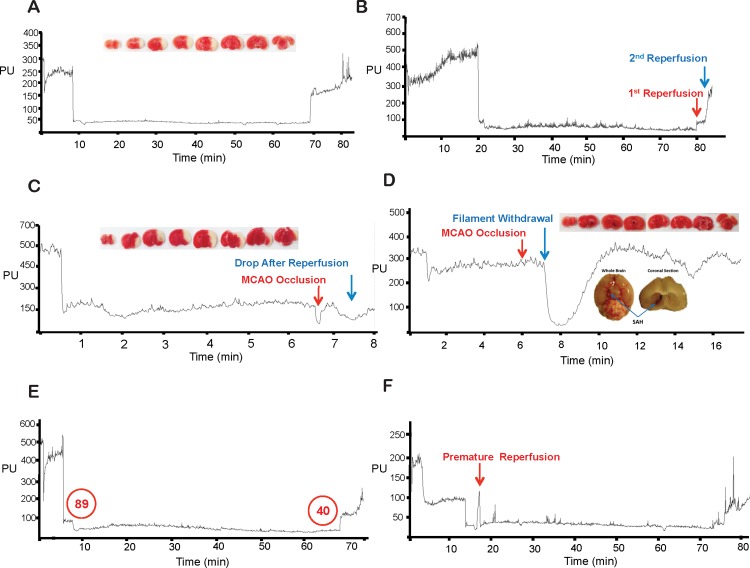
Representative laser-Doppler flowmetry traces of SAH and premature reperfusion (A) A typical representative laser-Doppler flowmetry during Koizumi’s method of middle cerebral artery occlusion (MCAO), during a 60 min occlusion. Insert: representative resulting ischemic lesion analysed by TTC staining. (B) A typical representative laser-Doppler flowmetry during Longa’s method of MCAO, during a 60 min occlusion. (C) Representative laser-Doppler flowmetry of subarachnoid haemorrhage (SAH) during the Koizumi method of MCAO, with resulting ischemic injury. (D) Representative laser-Doppler flowmetry of false-positive SAH. Inserts: image of the same animal, with a brain bleed resulting from vessel perforation, yet leading to no discernible ischemic injury. (E) Representative laser-Doppler flowmetry showing arbitrary units of CBF falling gradually over the course of a 60 min MCAO occlusion time, conducted via the Koizumi method. (F) Representative laser-Doppler flowmetry indicating premature reperfusion, due filament movement out of place, followed by immediate recovery of MCAO by repositioning the filament. Perfusion Units (PU) are arbitrary units of cerebral blood flow.

Although laser-Doppler provides only arbitrary readouts in CBF value during surgery, occlusion and reperfusion, its main usefulness not only lies in the identification of when the occluding filament is in the right location, but also in the identification of rare events such as possible SAHs and premature reperfusion during occlusion, which can assist with the correct removal of data as a result. As a guide for identification we have provided some examples of these events occurring in our own data sets (Fig 6). SAH was identified as previously described [25,41], via a distinct drop in CBF following removal of occluding filament, or a failure or reperfusion. In our study these cases were rare, with only 7 out of the 158 animals surviving to their collection time points requiring removal from the final data set due to possible SAHs occurring and only 1 out of the 158 removed due to possible premature reperfusion (Fig 6F). As a proof of principle that SAH can influence the lesion volumes, in the case of the animal represented in Fig 6C, a large lesion was apparent as a result of a possible SAH, despite this animal not undergoing any occlusion as they were in a designated sham group.

However, we also found that the laser-Doppler can provide false-positives of SAH. As represented in Fig 6D, there were two cases of MCAO occlusion failing to produce a distinct drop in CBF, but filament withdrawal causing a large drop in CBF, indicating SAH. Both these cases occurred in animals undergoing a SHAM surgery. In the case of the animal represented in Fig 6D, which was also undergoing a sham surgery, there was visible evidence of a blood clot having formed near the MCA, validating the Doppler result, but no ischemic lesion was visible, indicating the perforation of the vessel wall causing the blood clot may have repaired itself sufficiently to prevent any cellular injury. However, considering the case of the animal in Fig 6C, we immediately removed any data showing possible SAH in case lesion volume was inadvertently affected.

## Discussion

Owing to large differences in methodologies for the intraluminal filament method of MCAO in mice and rats, there is a lack of consensus on how best to perform surgery, which filaments to use for occlusion, the ideal time after reperfusion at which to analyse ischemic lesions, or how long occlusion periods should be in mice and rats, to create reproducible ischemic infarcts. In this study we manipulated these variables to produce consistent ischemic infarcts, the size of which could be altered by increasing or decreasing occlusion time. In the following we discuss these results in the context of wider literature.

### Surgical Experience Improves Surgical Outcome in the Intraluminal Filament Model of MCAO

Surgical skill, when performing intraluminal filament models of MCAO in rodents, is often cited as a major possible cause of inconsistency in resulting lesion volumes [17,42]. To reduce the impact of surgical experience on variability in this study, the surgeon (GM) was trained extensively over >100 surgeries, with guidance from two experienced practitioners (RT and AG), prior to gathering data. When mapping the progress of this training period, it was clear the ability to perform a successful surgery increased over time. Mortality caused by surgical error (S2 Table) reduced from 60.6% in an initial group of practice surgeries, to only 4.8% in a later group (S1 Table). We also attributed high mortality rates in our early study to the long (90 min) occlusion time we were initially using, shifting to a 60 min occlusion period as the longest utilised in this study. Mortality of animals 24 h post-surgery markedly decreased with practice (>80% down to <52%, S1 Table), and was similar to levels seen in other studies, which have been as high as 60–85% in mice [35,43,44]. Considering mice are more susceptible to occlusion time than rats, where a 90 min occlusion is common, this is not surprising [2]. Another important factor we noted was that the insertion distance of filaments, based on laser-Doppler guided placement, was highly consistent at 9.0 ± 0.5 mm from the bifurcation of the CCA, in line with other studies using similar age and weight C57BL/6 mice [45]. However, it is common to see an insertion distance of 11 mm [46–48] or insertion until ‘a mild resistance is felt’ [49], which we are concerned raises the possibility of inadvertent perforation of the ACA, increasing lesion volume and inconsistency in the model, a view shared by others [32].

As a result of undertaking the rigorous training progress, the number of mice required in this study was profoundly decreased, considering the markedly increased survival rate following extensive training (S1 Table). Of the 274 animals entered into the main study, only 8 out of 274 (2.9%), were deceased due to error during surgery, and only 16 out of 158 (10.1%) that survived to their designated times after reperfusion were removed from final analysis due to either surgical anomalies such as the movement of the filament during occlusion, possible SAH (Fig 6), or because they were outliers. This compares favourably to other studies, where as many as 30% of animals have been excluded from final analysis [50]. A particularly encouraging statistic was the 95.9% (47/49) survival rate in animals undergoing sham surgeries, indicating the vast majority of deaths post-surgery are likely due to the impact of the occlusion and resulting lesion, rather than surgery related injury.

A major factor contributing to the difficulty of surgery, particularly in mice that have a narrow surgical window compared to rats, is the anatomical differences between mice, even amongst the same strain. As two quantifiable examples, the occipital artery (OA) and superior thyroid artery (STA), each isolated and cauterised in our surgical procedure, can originate from several different structures. As reported by Chen et al., [8], the OA was originally described as bifurcating from the proximal segment of the ECA in mice. However, similar to their observations, we found the OA instead originated from the ICA, the proximal segment of the ECA and the bifurcation of the CCA 66.5%, 10% and 23.5% of the time, respectively (S3 Table). The STA on the other hand had a more consistent appearance, originating from the ICA, the proximal segment of the ECA and the bifurcation of the CCA 6.8%, 91.3% and 1.9% of the time. The impact of these differing origins on surgery is to increase the difficulty of their removal. For instance, an OA arising more distal to the bifurcation is likely in closer proximity to the vagus nerve, increasing the risk of inadvertent damage to this structure.

### Alterations to tip width of occluding filament did not affect lesion volume

It is apparent from previous studies that minor alterations in filament diameter can greatly affect resulting lesion volume and consistency in the mouse intraluminal MCAO. In one study, a large variation of 0.08 to 0.26 mm diameter filaments was employed on a body weight-to-diameter scale, with smaller mice undergoing occlusion with thinner filaments [45]. Employing this weight-to-diameter method resulted in consistent infarction in all mice. In another study, 8 filaments coated with poly-l-lysine to diameters between 110 and 180 μm were compared, and only the larger 170 and 180 μm (0.17 and 0.18 mm) diameter filaments provided consistent occlusion of the MCA, and the largest 180 μm thick filament provided a consistent lesion volume, with small standard error [51]. More recently however there has been an interchangeable use of slightly thicker 0.21 mm [33] and 0.23 mm [8,30] diameter silicone coated filaments produced by the Doccol company. We therefore analysed if the minor difference in diameter provided any difference in post-stroke measures following occlusion. We found no significant difference in lesion volume when comparing these two filaments at any time point after reperfusion, nor were there any differences in resulting CCAO, MCAO or reperfusion, indicating both diameter filaments occluded the MCA to the same reproducible degree. Survival post-surgery was also similar for both groups. Therefore, both 0.21 mm and 0.23 mm thick silicone coated filaments are suitable for use in the intraluminal filament method of MCAO in mice, within the body weight and age ranges used in this study.

### No changes in total ischemic lesion volumes were observed following varying times after reperfusion using TTC staining

Somewhat surprisingly, no significant differences in total lesion volume were observed when we measured the levels of ischemic lesions just 30 min and 4 h after reperfusion, compared to 24 h after reperfusion. This result is in contrast to a previous study in mice, where lesion was shown to evolve from 1.5 h to 24 h in mice [14], and also from 0 to 24 h using an *in vitro* TTC staining technique in rats [52].

However, Hatfield et al., showed that TTC staining using a perfusion technique could clearly identified lesions at 30 min after reperfusion that are not significantly different to those at 24 h in rats [29]. More recently, and also using an *in vivo* TTC-staining perfusion technique in rats, infarct areas were shown to be large immediately after reperfusion, but significantly decreased after 16h reperfusion, before significantly increasing once more by 24 h [52]. This is similar to our results with thin filaments, whereby we have a significantly smaller ischemic area at 12 h after reperfusion than at 30 min, which then increases again by 24 h. As Benedek et al., suggested, this could represent recovery of penumbra at 12 h [52], an observation also partially supported by our results showing fewer mice with a lack of staining in the thalamus and hippocampus by 12 h compared with earlier time points. Reversible injury following longer reperfusion has also been detected using TTC staining, suggesting some viable cells with perturbed mitochondrial activity, early after MCAO, may recover following longer periods of reperfusion [53].

Another more simple explanation for ischemic injury failing to increase over time in our study may be due to a rapid decline in survival post-12 h. It is entirely possible animals with larger lesions were amongst those less likely to survive, hence a lack of clear evidence for lesion and neurological deficit evolution at later time points after reperfusion.

In any case, our results, and those by others, question the reliability of the TTC stain to accurately determine the evolution of infarct during early time points after reperfusion, especially considering the stain may produce a false-positive detection of injury due to transient alterations in the ability of mitochondrial enzymes to metabolise TTC [52–55]. There is also a suggestion TTC may be unreliable at time after reperfusion longer than 24 h, considering later infiltration of healthy marcophages into infarcted tissue could increase the level of TTC staining [56].

Despite these qualms, a debate could be made that early, reversible cell death detectable by TTC staining, is a useful marker of regions likely to undergo degeneration [14], and there has been a number of studies that have validated TTC staining as a reliable tool for infarct assessment in comparison to other common methods such as Fluoro-Jade B, NeuN and cresyl violet staining, and light microscopic evaluation [14,54,57,58].

### Intraluminal filament MCAO with long coated filaments consistently causes ischemic injury in regions outside of the MCA territory

As expected, the striatum and cortex were the predominant brain regions effected during the course of MCAO evolution [2], and this was reflected in the largest areas of infarct consistently arising in sections corresponding to Bregma 0.0 to -1.0. However, with increasing occlusion time, we noticed an interesting trend in the hippocampus, where the dorsal and ventral hippocampus often lacked staining, suggesting ischemic injury, at earlier time points (8/18 and 9/18 respectively for all animals with 60 min MCAO via the Koizumi method, with thick filaments, analysed 4 h after reperfusion), yet tended to have little obvious hippocampal injury at 24 h (1/10 and 1/10 respectively for all animals with 60 min MCAO via the Koizumi method, with the thick filaments, after 24 h after reperfusion).

A similar trend was seen in the thalamus with 30 min (2/7), 4 h (7/18), 12 h (1/5) and 24 h (1/10) animals showing ischemic lesions in this territory (following a 60 min MCAO via the Koizumi method with the thick filaments), suggesting there may be reversible injury in these areas, when analysed by TTC staining. However, the proportion of animals with ischemic injury in the amygdala increased with increasing time after reperfusion, with 30 min (2/7), 4 h (8/18), 12 h (5/5) and 24 h (9/10) animals showing lesions in this territory (following 60 min MCAOs via the Koizumi method with the thick filaments). No ischemic lesions were seen in the any of these areas after shorter durations occlusions (15 and 30 min), indicating the extent of ischemic injury was dependent on duration of occlusion, as confirmed in other studies [19].

Despite the fact the hippocampus, thalamus and amygdala are not supplied by the MCA, it is not uncommon for these regions to show evidence of ischemic injury following MCAO [19,44,45,59], for several reasons. First and foremost, C57BL/6 mice may be more susceptible to variation in intraluminal filament MCAO compared to other strains owing to a common lack of patency in posterior communicating arteries (PComA) [31,44]. Up to 30% of C57BL/6 may be deficient in both PComAs, with perhaps only 10% having both [19]. The consequence of missing PComAs is that territory supplied by the posterior communicating artery (PCA) will also experience a lack of blood flow when an intraluminal filament is present, as a lack of a PComA will prevent collateral blood supply to this area during occlusion [19,44]. Considering the PCA supplies blood to the hippocampus and thalamus, these areas may therefore experience ischemia during occlusion. Even with shorter coated filaments it is impossible to circumvent the issue of poorly developed or absent PComAs, as in this case blood supply to the ipsilateral ACA, and therefore PCA, will still be blocked.

Second, blood supply may also be inadvertently blocked to the anterior choroidal artery (AChA), ventral thalamic artery (VTA) and hypothalamic artery (HTA, Fig 1). This is especially likely with longer coated filaments, as the AChA, VTA and HTA branch from and are supplied by the internal ICA (Fig 1) [23,60]. Despite using longer coated filaments, we only saw evidence of hypothalamic lesions in one mouse, across all groups, which is less than has been reported previously [19]. As mentioned above however, we did see lesions in the thalamus, which may not only be due absent PComAs, but perhaps also by blockage of the VTA. Interestingly, the likelihood of injury in the thalamus did appear to decrease when shorter coated filaments were used.

Finally, regions outside of the MCA territory may be susceptible to collateral damage from the effects of a variety of pathophysiological mechanisms of ischemic injury including excitotoxicity, spreading depression, reactive oxygen species, inflammation and apoptosis [36].

As mentioned above, after extended periods of reperfusion, we saw increased ischemic lesions in the amygdala, which is supplied by the AChA [61]. As with the thalamus, the likelihood of injury to the amygdala seemed lower in animals occluded with shorter coated filaments after 4 h reperfusion. These results make it tempting to speculate that the coating length of filaments is a major determinant of ischemic injury to areas outside of the MCA territory in the intraluminal model, a hypothesis which is supported by evidence that the distance between the MCA and the PCA is only 1.75 ± 0.18 mm in C57BL/6 mice [62]. To the best of our knowledge however, the relative distances of the MCA, AChA, VTA and HTA in mice is yet to be accurately measured [61]. Defining these distances may aid in determining the optimal length of filament coating to prevent ischemic lesions occurring in areas provided by these arteries.

### Ischemic injury increases sharply between 30 and 45 min of occlusion

Studies using the intraluminal filament model of MCAO generally utilise one distinct occlusion period which can range from brief (15 min) to longer (180 min, or permanent). Despite this, lesion volumes have varied greatly from lab to lab, with some producing much larger lesions volumes than others, even with the same occlusion period [2]. In our study few ischemic lesions were noticeable following minor (15 min) periods of occlusion when analysed by TTC at 4 h, and only inconsistent, small lesions were visible following 24 h reperfusion after 15 min occlusion in the striatum and cortex. However, with increasing occlusion periods, the increase in lesion volume was remarkably consistent, until 45 min, wherein lesion volume appeared to peak, with no statistically significant difference between the overall lesion volume generated following a 45 min or 60 min occlusion. This does not necessarily mean 45 min is the ‘threshold’ level of occlusion in mice, as 2 h occlusion has in the past been shown to significantly increase lesion volume over a 1 h occlusion [47], however it is a fair indication there is a considerable jump in lesion volume between 30 min and 45 min occlusion, suggesting >30 min of occlusion dramatically increases ischemic injury in the mouse.

Others have found a steep difference in lesion volume between only 15 and 30 min of occlusion, when performing a similar time course, although these results were obtained with different filament properties, entry via the ECA rather than the CCA, and older mice than in our model [19]. Remarkably in the rat, lesion volume may not peak until after 2–3 h [15], or perhaps even 3–4 h of occlusion [63]. Together these results indicate a much greater susceptibility of mice to occlusion time than rats.

### The Longa and Koizumi methods of intraluminal filament MCAO do not produce differences in lesion volume in mice at 4 h reperfusion

To the best of our knowledge, only two studies exist directly comparing the Longa and Koizumi methods of intraluminal filament MCAO in rodents, despite widespread use of both techniques. The first, from Laing et al., in rats, illustrated that the method of Longa was shown to be unreliable in reproducibly inducing infarct, compared to the Koizumi method [22]. This was thought likely due to the properties of the filament employed in Longa’s method, which were more likely to induce vessel perforation during insertion [22]. A more recent study in mice compared the two methods, but employed a consistent 180 μm silicone-tipped filament when performing both methods [64]. Contrary to the study of Laing, this study concluded that the Koizumi method produced a higher mortality rate after 24 h reperfusion, but the lesion volumes did not differ at 24 h.

Utilising both short and long silicone coated filaments, it was interesting to note in our study that reperfusion was significantly increased by the Longa method in comparison to the Koizumi method, using both calculations of reperfusion, a trend which was also found by Smith et al., [64]. Smith et al., reached the conclusion that the Longa method is superior to the Koizumi method for short and long-term study ischemic stroke in mice partially based on differences in reperfusion, but also partially due to increased survival at 24 h and increased lesion volumes following the Longa method at 1 week post-occlusion, compared to the Koizumi method. However, due to a lack of significant differences in lesion volume between the two models, albeit at 4 h post-reperfusion, we hesitate to draw the same conclusion. In fact, the greater death rate following the Koizumi surgeries in the study by Smith et al., could be used as an argument for a more successful ischemic injury in this group of animals, which would be likely to impact resulting lesion volumes at both 24 h and 7 d, as animals with larger lesions may not survive to these time points. Further research is required to compare and contrast the usefulness of these two models under different scenarios, utilising different filaments, different occlusion periods, different analysis times after reperfusion, and different techniques for cell death assessment.

### Relative CCAO, MCAO and reperfusion laser-Doppler measurements do not correlate with lesion volume, but can identify rare cases of SAH and premature reperfusion

Laser-Doppler measurement has long been utilised as a tool to assist with intraluminal filament surgeries as it offers a simple, continuous, instantaneous, inexpensive and non-invasive way to assess relative CBF alterations in specific brain regions. Although absolute values obtained from laser-Doppler instruments are arbitrary, relative changes in CBF correlate well with other methods of CBF detection such as autoradiography [65]. When laser-Doppler data is reported, it is often accompanied by a statement that animals failing to register a 70% drop from baseline CBF, following intraluminal filament occlusion of the MCAO, are excluded from final datasets [66,67]. In some cases this cut-off has been 60% [17,68–70], 80% [71] or as high as 85% [14], but 70% has been a popular choice, in both mouse and rat studies [37–39].

The 70% figure perhaps derives from studies aiming to determine ‘thresholds’ for CBF, below which tissue will become irreversibly injured during MCAO [72,73]. For instance, using the [^14^C]iodoantipyrine (IAP) autoradiographic technique, changes in local CBF were mapped to the probably of infarct in a rat model of intraluminal filament MCAO [74,75]. It was determined that decreases in local blood flow of 0–20% gave a 96% probability of infarction, leading to irreversibly injured core areas of infarct. Even minor differences in this level substantially altered the probability of infarction, with penumbral regions (surrounding the core) only experiencing decreases in CBF to 20–40% of control.

However, the applicability of these thresholds to intraluminal filament MCAO in the mouse, using laser-Doppler to derive relative changes in CBF, is unclear. Early papers using laser-Doppler flowmetry in the intraluminal filament technique of MCAO did not appear to exclude animals failing to meet thresholds [25,41,45,76], nor do some more recent papers [77]. Furthermore, to the best of our knowledge there is no statistical comparison between laser-Doppler measurements of MCAO and resulting lesion volume in mice that suggests differences in the percentage drop in CBF following MCAO leads to alterations in lesion volume. Therefore there is no statistical support for the exclusion of animals with a relative change in CBF of <70% in the MCA territory following MCAO, calculated using laser-Doppler measurements. There is one relevant study in rats suggesting perfusion deficits, measured by laser-Doppler in the territory of the MCA-ACA leptomeningeal branches, but not the MCA territory, may be a useful predictor of resulting infarct volume and neurological deficit [78]. A lack of these correlations in mice may be the cause of the current varied approaches to animal exclusion on the basis of laser-Doppler measurements.

Considering standardising approaches to modelling MCAO between labs may help improve reproducibility, we correlated our laser-Doppler measurements of CCAO, MCAO and reperfusion, to the resulting lesion volumes for each animal in our study. Although the majority of animals in the study did experience a relative CBF drop of >70% of arbitrary laser-Doppler values following MCAO (138/153 (87.9%)), we found no statistical evidence of a correlation between CBF drop following MCAO and lesion volume (Table 4) to support the removal of data from the small proportion of animals who fell outside of the 70% cut-off (15/153 (9.8%) between 60–70% and 4/153 (2.6%) <60%. We also did not find any statistical evidence of correlation between CCAO or reperfusion, calculated by either method, to resulting lesion size. These findings are in line with the study of Taguchi et al., who found no correlation of relative CBF alterations following MCA coagulation, analysed by laser-Doppler (at the same coordinates used in this study), to resulting lesion volume [79]. The study of Hedna et al., also failed to find a correlation between CBF alterations following CCAO or MCAO and resulting lesion volumes, although animals failing to register >80% CBF reduction during ischemia were removed from analysis [71].

There are several alternative reasons to explain why the relative changes in CBF did not correlate to total lesion volume in our study;

1 The placement of the probe is important, as even minor differences will alter the cerebral territory being sampled for measurement of CBF, and can markedly change the level of arbitrary CBF values [65]. Considering sites measuring CBF over the parietal cortex can sample a mixture of MCA and ACA flow [80], this could be a common problem. These issues are compounded by the small sample volume of the laser probe [81] which means a single probe can only sample discrete areas undergoing ischemia and cannot provide information about CBF in other regions.

A wide variety of placement points, relative to Bregma, have been used in different studies in mice such as 6 mm lateral and 2 mm posterior from Bregma [82], 1 mm caudal and 3 mm lateral from bregma [83], 0.5 mm posterior and 4 mm lateral from bregma [84] and 1 mm posterior and 5 mm lateral to bregma [71]. These differing locations ultimately sample either distal or proximal segments of the MCA [24], and therefore may provide differing relative measurements of alterations in local CBF, following MCAO. Minor differences in probe position have been shown to influence relative CBF changes, following MCAO, in rats [78,85]. In this study we did not find significant correlations between relative alterations in CBF and resulting lesions following MCAO when placing the laser-Doppler probe at Antero-Posterior (AP) ~-1.0 and Medio-Lateral (ML) ~3.0 from bregma.

2 Laser-Doppler measurements are highly sensitive to fluctuation from mechanical movement around the probe, which could immediately impact relative changes in CBF over time, especially when probes are adhered to the skull using simple surgical glue (as it was in our study), rather than being attached using burr holes [85].
3 Laser-Doppler measurements, because they are continuous, can suffer from a phenomena known as vasomotion, whereby oscillating CBF can continuously fluctuate relative CBF values [65].
4 Differences in intra and interspecies microvasculature [86], perhaps as a result of aging [87], may create differences in collateral flow to MCA supplied territories, altering resulting relative CBF changes when the CCA and MCAO are occluded.

Finally, in addition to our lack of significant correlations, we noted an interesting phenomenon indicating a reduction in CBF over the course of occlusion, significantly so after a 45 min occlusion (Table 2), compared to shorter occlusion times, but the trend was also evident in all groups with occlusion longer than 15 min, compared to 0 min sham occlusion. Despite this declining CBF, all animals included in our study had a sharp increase in CBF following filament removal, although the degree of increase varied widely. It is unclear whether this reduction over time represents a real physiological effect, or if it is mechanical in origin, such as the contact between the laser-Doppler probe and the skull weakening throughout the surgery and occlusion due to accidental movement of the probe by the surgical operator. There has been one other report of laser-Doppler data in mice discussing the phenomenon of increased cerebral hypoperfusion following longer periods of occlusion [19], although the suggested reason for this effect was declining blood pressure and neurochemical events associated with ischemia, rather than mechanical.

In any case, if mechanical rather than physiological, this decrease in CBF over occlusion may impact the possible level of reperfusion compared to the original baseline measurement, necessitating the need for the normalised measurement of reperfusion employed in our study (Fig 2). In fact, it was striking to note the large differences in values when comparing the calculation of reperfusion via either our modified fold change calculation or by the traditional percentage based calculation. When calculating by the traditional method, reperfusion was clearly lower in comparison to sham groups, following occlusion, whereas reperfusion was not significantly different to sham groups by the modified method. If the drop in CBF over time of occlusion is mechanical, as suggested above, this means the traditional measure of reperfusion may be falsely indicating the exact level of reperfusion. However, as evidence to the contrary, it was clear that reperfusion in Longa groups was essentially back to 100% of baseline even when calculating by the traditional method, whereas the Koizumi groups were consistently lower than this, indicating there is a definitive increase in reperfusion following Longa surgeries compared to Koizumi surgeries, and supporting evidence suggesting the Koizumi method does not produce full reperfusion via the contralateral hemisphere following removal of the intraluminal filament [64].

In summary, we do not find statistical evidence to support the exclusion of data on the basis of a 70% cut-off following MCAO, calculated using a laser-Doppler, in mice. A recent study, in rats, indicated correlations between laser-Doppler measurements and lesion volume when sampling territories outside of those supplied by the MCA, but not the MCA [78], suggesting it would be interesting to validate the role of the laser-Doppler as a predictive tool in mice further. At the very least we suggest correlation analysis should be performed between laser-Doppler data and lesion data, before excluding animals, rather than employing arbitrary cut-off points prior to beginning a study. This conclusion is in line with other recent findings suggesting that there is little benefit when using the laser-Doppler, regardless of surgical expertise, except for perhaps slightly decreasing the number of animals required in each study by decreasing the coefficient of variance in resulting lesion volume when laser-Doppler measurements are available to the surgeon [17]. We do however find real-time laser-Doppler recordings are useful for the identification of SAH and premature reperfusion, and for assisting with correct placement of the intraluminal filament at the MCA.

### Age, starting body weight, body weight loss, long-term survival and many other factors may influence lesion volume

Although we have analysed and discussed several key variables which can affect lesion volume in the intraluminal MCAO model of stroke including the choice of intraluminal filament, occlusion time and reperfusion time, we note there are many other variables which may alter the magnitude of resulting lesions. These variables include, but are not limited to, body temperature during and after surgery, choice of anaesthetic, starting body weight, body weight loss following MCAO, age, sex, and strain of mice. In this study we kept these variables consistent between groups, however we correlated starting body weight, body weight loss and age to resulting lesion volumes.

Despite the fact age is a primary risk factor for stroke in humans, animal studies rarely utilise aged animals, largely due to pragmatic issues such as cost and surgical difficulty (older animals tend to have more fat surrounding the CCA, ECA and ICA making artery isolation more difficult [88]). As such, in most studies animals are within a similar age range to that employed here (~8–12 weeks old). It has been shown however that aged animals can have significantly larger injuries in the striatum and neocortical areas, but significantly smaller hippocampal injuries compared to younger animals, following intraluminal filament MCAO [21]. In our study we saw no correlation between the age of mice and resulting lesion size, although this is likely due to the narrow age range used (Table 4).

It has been logically suggested that matching the body weight of animals to the width of the occluding filament may improve the reproducibility of lesion [89], presumably due to the assumption arterial width differs in different weight animals [88]. As mentioned earlier, a linear relationship between body weight and filament diameter required for occlusion has been observed [45] and previous research indicated a minimum filament width of 170–180 μm can produce reproducible lesion volumes in mice [54].

In this study we found no correlation between the starting body weight of animals and the resulting lesion size, using either 0.21 or 0.23 mm width filaments, in 16 out of 17 groups (Table 4). This suggests that 0.21 mm width sutures are wide enough to produce reproducible lesion volumes in the range of body weights and ages used in this study and that 0.23 mm width sutures were not so wide that they caused inconsistency due to possible vessel damage. However, the weight and age ranges of animals analysed in this study were relatively narrow and matching body weight to filament width may become more important in younger/older and/or lighter/heavier animals than those used in this study, or when employing thinner and thicker filaments than the two trialled here. It would be useful to test these hypotheses in future.

There was however a weak correlation between starting body weight and lesion volume in the group of animals undergoing a 60 min MCAO following the Koizumi method with thick sutures, when analysed at 4 h post-MCAO and interestingly this group was one with an appreciable number of animals (n = 20). We are unsure why this correlation occurred. It is worth mentioning the emerging understanding of an “obesity paradox” from recent clinical stroke data, whereby although obesity is a risk factor for stroke, there is a possible survival benefit for overweight individuals post-stroke [90]. We find it difficult however to apply this idea to the current study as the survival rate at 4 h post-surgery is relatively high. In any case, it would certainly be interesting to explore this effect further and at the very least our data reinforces the importance of ensuring the starting body weight of animals is consistent across experimental groups.

There is some evidence to suggest that body weight loss, post-MCAO, may be predictive of lesion volume. One study found that gross body weight loss post-surgery is significantly increased in mice undergoing occlusion, compared to sham animals at 2, 4, 7, 14, 21 and 28 days post-surgery, although surprisingly sham-operated animals did not lose body weight after surgery [30], as they did in our study. In another study mice undergoing 2 h or permanent occlusions were shown to have significantly larger body weight loss than sham operated animals, or animals undergoing 1 h occlusion, 7 days after MCAO, compared with 24 h after MCAO [91]. These data are corroborated by evidence that body weight loss correlates with lesion volume at 3 d, but not 24 h or 7 d, post MCAO [40]. In our study we found body weight loss significantly increased from 4 h to 24 h post-MCAO (Fig 3), however we did not find a correlation between body weight loss and lesion volume (Table 4), nor were there any differences in body weight loss between the sham and occlusion groups. This is in line with the aforementioned observations of others, which suggest body weight correlates at only 3 d post-MCAO.

One of the limitations of our work is that survival, lesion volume and neurological deficits were not assessed beyond 24 h. In studies assessing lesion volume at 24 h, 3 d and 7 d, lesion volume was shown to peak at 24 h post-reperfusion in mice [14]. That being said, it would be useful to measure lesion volume beyond 24 h when investigating possible neuroprotective agents, which may have delayed efficacy. In order to do so it would be beneficial to increase the survival rate from those we have observed here (<40% in our 60 min occlusion, 24 h reperfusion groups). This may be possible with the use of prophylactic antibiotics, as others have shown [30]. Alternatively, as we have observed, decreasing the occlusion period increases the survival rate, despite reducing the lesion volume.

A second limitation of this work was the use of only one method, TTC, to analyse lesion volume. As mentioned earlier, TTC staining provides a comparable measure of lesion volume to other techniques, however it may not be reliable beyond 24 h as infiltrating immune cells may obscure the real magnitude of lesion [81]. Any studies assessing outcomes beyond 24 h should therefore consider using alternative measures of lesion volume.

## Conclusion

We conclude that any investigator attempting intraluminal filament MCAO surgery must undertake a rigorous training process in order to establish a successful, reproducible technique. Progress can be mapped through reductions in common surgical errors and assessments of survival over time. Secondly, both the Koizumi and Longa method are capable of providing reproducible ischemic lesions of similar magnitude, the volume of which can be manipulated by altering occlusion time. The Koizumi method is perhaps simpler surgically, but the Longa method provides significantly greater reperfusion post-occlusion. Finally, laser-Doppler measurements should be carefully examined to determine if CCAO, MCAO, alterations in CBF over occlusion time, or reperfusion are altering the volume of ischemic lesions, prior to rejecting data based on pre-determined cut-off points.

## Supporting Information

S1 Fig Position of each coronal level and structures within them, relative to Bregma and example of lesion calculation via the Swanson formula. (TIFF)

S2 Fig Ipsilateral lesion volume, relative to Bregma, for each experiment, at all eight coronal levels. (TIFF)

S1 Table Example of improvement in survival of mice with increased surgical experience of operator, when performing the intraluminal filament middle cerebral artery occlusion via the Koizumi method. (PDF)

S2 Table Examples of likely causes of death from intraluminal filament MCAO surgery via the Koizumi method, both during and post-surgery, by an inexperienced operator. (PDF)

S3 Table The position of origin for occipital and superior thyroid arteries, in C57BL/6 mice used in this study. (PDF)

S4 Table Survival statistics for mice undergoing the reperfusion time course (Fig 3) following 60 min of intraluminal filament MCAO via the Koizumi method. (PDF)

S5 Table Survival statistics for mice undergoing the occlusion time course (Fig 4) at 4 h and 24 h post-reperfusion after intraluminal filament MCAO via the Koizumi method. (PDF)

S6 Table Survival statistics for mice undergoing the Longa and Koizumi surgical comparison (Fig 5) at 4 h post-reperfusion after intraluminal filament MCAO. (PDF)

S7 Table Ischemically injured regions following MCAO for all groups analysed during this study. (PDF)

S8 Table Raw data for Fig 3. (XLSX)

S9 Table Raw data for Fig 4. (XLSX)

S10 Table Raw data for Fig 5. (XLSX)

S1 Video Intraluminal filament middle cerebral artery occlusion in C57/BL6 mouse (Longa and Koizumi methods). (MP4)
