# Supplementary figures and images for: A Comparative Study of Variables Influencing Ischemic Injury in the Longa and Koizumi Methods of Intraluminal Filament Middle Cerebral Artery Occlusion in Mice
Source: PLoS One. 2016 Feb 12;11(2):e0148503. doi: 10.1371/journal.pone.0148503 (PMC4752454; doi:10.1371/journal.pone.0148503)

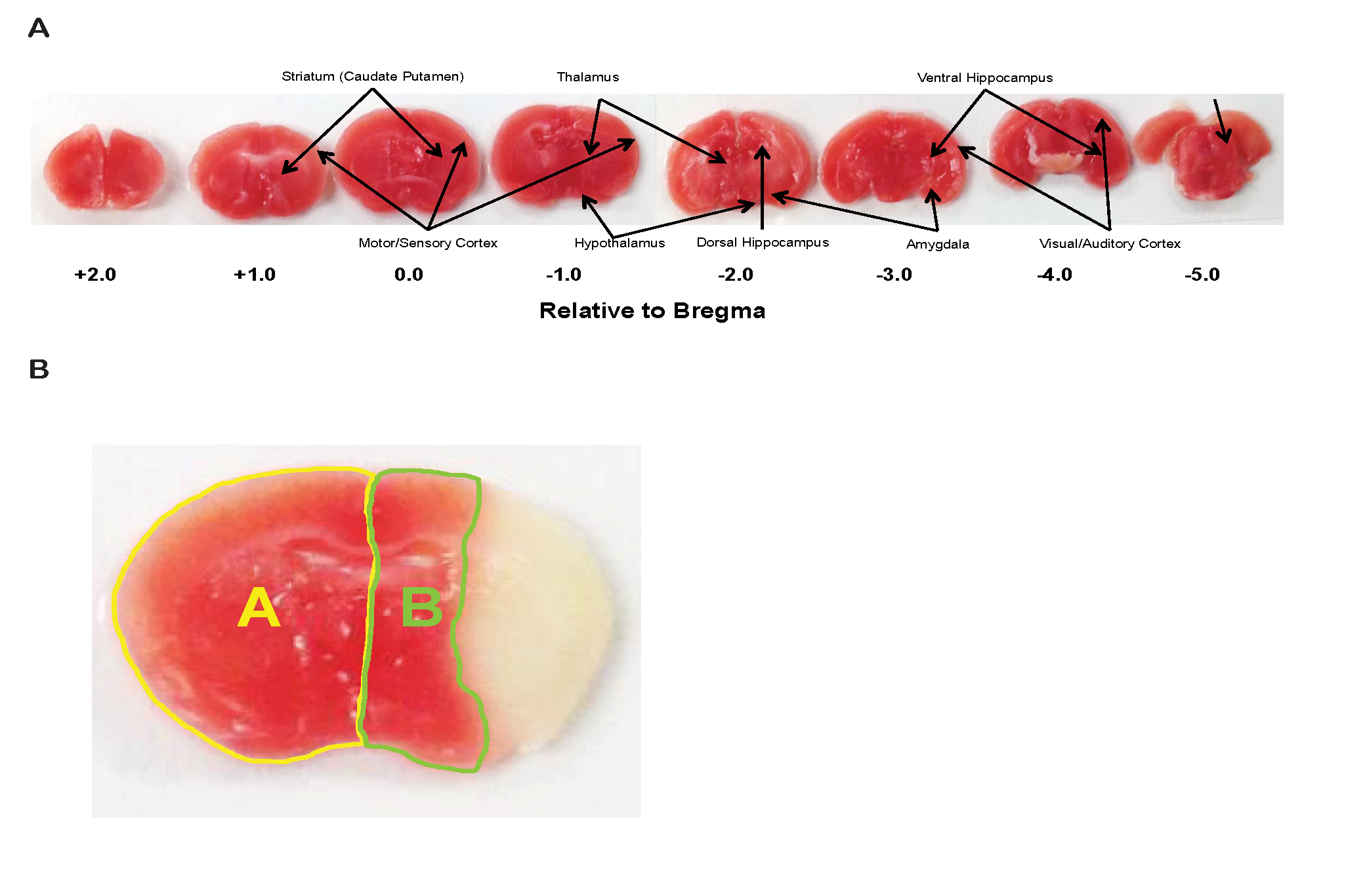

Supplement: S1 Fig — (TIFF) [file pone.0148503.s001.tiff]

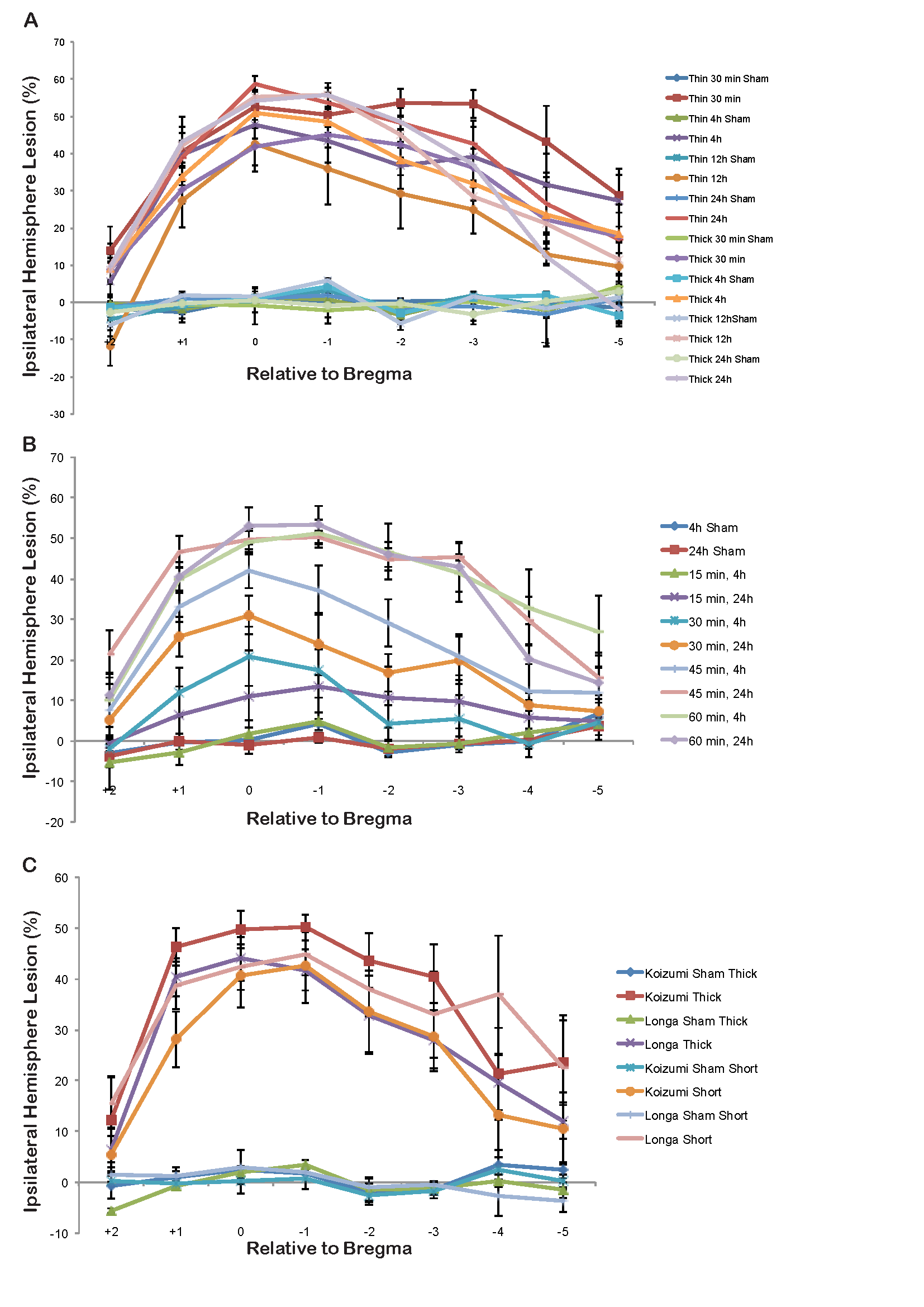

Supplement: S2 Fig — (TIFF) [file pone.0148503.s002.tiff]
