# Supplementary material for: A Comparative Study of Variables Influencing Ischemic Injury in the Longa and Koizumi Methods of Intraluminal Filament Middle Cerebral Artery Occlusion in Mice
Source: PLoS One. 2016 Feb 12;11(2):e0148503. doi: 10.1371/journal.pone.0148503 (PMC4752454; doi:10.1371/journal.pone.0148503)
Supplement: S1 Table — (PDF) [file pone.0148503.s003.pdf]

Supplementary Table 1. Example of improvement in survival of mice with increased surgical experience of operator, when performing the intraluminal filament middle cerebral artery occlusion via the Koizumi method

Surgical Group	n
90 min occlusion	33
90 min occlusion	34
60 min occlusion	42

Deceased During Surgery (%)	Deceased Post-Operatively (0-24h) (%)	Survival (24h) (%)
60.6	21.2	18.2
41.2	14.7	44.1
4.8	47.6	47.6
