# Supplementary material for: A Comparative Study of Variables Influencing Ischemic Injury in the Longa and Koizumi Methods of Intraluminal Filament Middle Cerebral Artery Occlusion in Mice
Source: PLoS One. 2016 Feb 12;11(2):e0148503. doi: 10.1371/journal.pone.0148503 (PMC4752454; doi:10.1371/journal.pone.0148503)
Supplement: S2 Table — (PDF) [file pone.0148503.s004.pdf]

Supplementary Table 2. Examples of likely causes of death from intraluminal filament MCAO surgery via the Koizumi method, both during and post-surgery, by an inexperienced operator

Surgical Group	90 min occlusion	90 min occlusion
**n**	33	34
**ICA Bleed**	2	0
**ECA Bleed**	6	0
**CCA Bleed**	0	0
**OA Bleed**	2	0
**Clamp Removal Bleed**	3	2
**Unidentified Bleed**	3	9
**Nerve Damage**	6	2
**Muscle Damage**	0	1
**Incorrect Ketamine Administration**	0	1
**Unidentified Cause**	5	3
**SAH**	0	1

60 min occlusion
42
0
0
2
0
0
4
2
1
1
12
0
