# Supplementary material for: A Comparative Study of Variables Influencing Ischemic Injury in the Longa and Koizumi Methods of Intraluminal Filament Middle Cerebral Artery Occlusion in Mice
Source: PLoS One. 2016 Feb 12;11(2):e0148503. doi: 10.1371/journal.pone.0148503 (PMC4752454; doi:10.1371/journal.pone.0148503)
Supplement: S3 Table — (PDF) [file pone.0148503.s005.pdf]

Supplementary Table 3. The position of origin for occipital and superior thyroid arteries, in C57BL/6 mice used in this study

Artery Position	Occipital Artery	Superior Thyroid Artery
**ICA**	184/310 (66.5%)	21/310 (6.8%)
**ECA**	31/310 (10%)	283/310 (91.3%)
**Bifurcation**	73/310 (23.5%)	6/310 (1.9%)
