# Supplementary material for: A Comparative Study of Variables Influencing Ischemic Injury in the Longa and Koizumi Methods of Intraluminal Filament Middle Cerebral Artery Occlusion in Mice
Source: PLoS One. 2016 Feb 12;11(2):e0148503. doi: 10.1371/journal.pone.0148503 (PMC4752454; doi:10.1371/journal.pone.0148503)
Supplement: S4 Table — (PDF) [file pone.0148503.s006.pdf]

Supplementary Table 4. Survival statistics for mice undergoing the reperfusion time course (Fig. 3) following 60 min of intraluminal filament MCAO via the Koizumi method

Recovery Time (h)	Filament	n	Deceased During Occlusion	Deceased Post-Occlusion	Survival to Collection	Removed Due to Possible SAH or Filament Movement
0.5	Sham Thin	3	0/3 (0%)	0/3 (0%)	3/3 (100%)	0/3 (0%)
	Sham Thick	3	0/3 (0%)	0/3 (0%)	3/3 (100%)	0/3 (0%)
	Thin	9	1/9 (11%)	1/8 (12.5%)	7/8 (87.5%)	0/8 (0%)
	Thick	8	1/8 (12.5%)	0/7 (0%)	7/7 (100%)	0/7(0%)
4	Sham Thin	3	0/3 (0%)	0/3 (0%)	3/3 (100%)	0/3 (0%)
	Sham Thick	3	0/3 (0%)	0/3 (0%)	3/3 (100%)	0/3 (0%)
	Thin	15	3/15 (20%)	3/12 (25%)	9/12 (75%)	1/9 (11.1%)
	Thick	11	1/11 (9.1%)	3/10 (30%)	7/10 (70%)	0/7 (0%)
12	Sham Thin	5	0/5 (0%)	0/5 (0%)	5/5 (100%)	1/5 (20%)
	Sham Thick	5	0/5 (0%)	0/5 (0%)	5/5 (100%)	2/5 (40%)
	Thin	16	1/16 (6.3%)	6/15 (40%)	9/15 (60%)	3/9 (33%)
	Thick	6	0/6 (0%)	1/6 (16.7%)	5/6 (83.3%)	0/5 (0%)
24	Sham Thin	4	0/4 (0%)	0/4 (0%)	4/4 (100%)	1/4 (25%)
	Sham Thick	4	0/4 (0%)	1/4 (25%)	3/4 (75%)	0/3 (0%)
	Thin	19	3/19 (15.8%)	10/16 (62.5%)	6/16 (37.5%)	0/6 (0%)
	Thick	21	2/21 (9.5%)	14/19 (73.7%)	5/19 (26.3%)	0/5 (0%)
