# Supplementary material for: A Comparative Study of Variables Influencing Ischemic Injury in the Longa and Koizumi Methods of Intraluminal Filament Middle Cerebral Artery Occlusion in Mice
Source: PLoS One. 2016 Feb 12;11(2):e0148503. doi: 10.1371/journal.pone.0148503 (PMC4752454; doi:10.1371/journal.pone.0148503)
Supplement: S5 Table — (PDF) [file pone.0148503.s007.pdf]

Supplementary Table 5. Survival statistics for mice undergoing the occlusion time course (Fig. 4) at 4 h and 24 h post-reperfusion after intraluminal filament MCAO via the Koizumi method

Occlusion time (min)	Recovery time (h)	n	Deceased During Occlusion	Deceased Post Occlusion	Survival to Collection	Removed Due to Possible SAH or Filament Movement	Outlier Removed
0	4	3	0/3 (0%)	0/3 (0%)	3/3 (100%)	0/3(0%)	0/3 (0%)
	24	4	0/4 (0%)	1/4 (25%)	3/4 (75%)	0/3 (0%)	0/3 (0%)
15	4	7	1/7 (14.3%)	1/6 (16.7%)	5/6 (83.3%)	0/5 (0%)	0/5 (0%)
	24	6	0/6 (0%)	0/6 (0%)	6/6 (100%)	1/6 (17%)	1/5 (20%)
30	4	6	0/6 (0%)	1/6 (16.7%)	5/6 (83.3%)	0/5 (0%)	0/5 (0%)
	24	14	2/14 (14.3%)	7/12 (58.3%)	5/12 (41.7%)	0/5 (0%)	1/5 (20%)
45	4	7	0/7 (0%)	1/7 (14.3%)	6/7 (85.7%)	1/6 (17%)	0/5 (0%)
	24	13	1/13 (7.7%)	6/12 (50%)	6/12 (50%)	1/6 (17%)	0/5 (0%)
60	4	13	2/13 (15.4%)	2/11 (18.2%)	9/11 (81.8%)	2/9 (22%)	0/7 (0%)
	24	14	1/14 (7.1%)	8/13 (61.5%)	5/13 (38.5%)	0/5 (0%)	0/5 (0%)
