# Supplementary material for: A Comparative Study of Variables Influencing Ischemic Injury in the Longa and Koizumi Methods of Intraluminal Filament Middle Cerebral Artery Occlusion in Mice
Source: PLoS One. 2016 Feb 12;11(2):e0148503. doi: 10.1371/journal.pone.0148503 (PMC4752454; doi:10.1371/journal.pone.0148503)
Supplement: S6 Table — (PDF) [file pone.0148503.s008.pdf]

Supplementary Table 6. Survival statistics for mice undergoing the Longa and Koizumi surgical comparison (Fig. 5) at 4 h post-reperfusion after intraluminal filament MCAO

Method	Filament	n	Deceased During Occlusion	Deceased Post-Occlusion	Survival to Collection	Removed Due to Possible SAH or Filament Movement
Koizumi	Sham Thick	3	0/3 (0%)	0/3 (0%)	3/3 (100%)	0/3 (0%)
	Sham Short	3	0/3 (0%)	0/3 (0%)	3/3 (100%)	0/3 (0%)
	Thick	11	2/11 (18.2%)	2/9 (22.2%)	7/9 (77.8%)	1/7 (14%)
	Short	6	0/6 (0%)	1/6 (16.7%)	5/6 (83.3%)	0/5 (0%)
Longa	Sham Thick	3	0/3 (0%)	0/3 (0%)	3/3 (100%)	0/3 (0%)
	Sham Short	3	0/3 (0%)	0/3 (0%)	3/3 (100%)	0/3 (0%)
	Thick	8	2/8 (25%)	1/6 (16.7%)	5/6 (83.3%)	0/5 (0%)
	Short	7	0/7 (0%)	2/7 (25%)	5/7 (62.5%)	0/5 (0%)
