# Supplementary material for: A Comparative Study of Variables Influencing Ischemic Injury in the Longa and Koizumi Methods of Intraluminal Filament Middle Cerebral Artery Occlusion in Mice
Source: PLoS One. 2016 Feb 12;11(2):e0148503. doi: 10.1371/journal.pone.0148503 (PMC4752454; doi:10.1371/journal.pone.0148503)
Supplement: S7 Table — (PDF) [file pone.0148503.s009.pdf]

Supplementary Table 7. Ischemically injured regions following MCAO for all groups analysed during this study

Surgical Group	Cortex	Striatum	Dorsal Hippocampus	Ventral Hippocampus	Thalamus	Hypothalamus	Amygdala	Colliculus
Thin 30 min recovery	7/7	7/7	6/7	6/7	5/7	1/7	2/7	2/7
Thin 4h recovery	8/8	8/8	4/8	4/8	4/8	0/8	4/8	1/8
Thin 12h recovery	6/6	6/6	1/6	1/6	2/6	0/6	2/6	1/6
Thin 24h recovery	6/6	6/6	2/6	2/6	2/6	0/6	6/6	1/6
Thick 30 min recovery	7/7	7/7	2/7	2/7	2/7	0/7	2/7	1/7
Thick 4h recovery	7/7	7/7	2/7	4/7	2/7	0/7	2/7	0/7
Thick 12h recovery	5/5	5/5	1/5	3/5	1/5	0/5	5/5	1/5
Thick 24h recovery	5/5	5/5	0/5	0/5	0/5	0/5	5/5	0/5
15 min occlusion, 4h recovery	3/5	0/5	0/5	0/5	0/5	0/5	0/5	0/5
15 min occlusion, 24h recovery	3/5	2/5	0/5	0/5	0/5	0/5	0/5	0/5
30 min occlusion, 4h recovery	3/5	2/5	0/5	0/5	0/5	0/5	0/5	0/5
30 min occlusion, 24h recovery	5/5	5/5	0/5	0/5	0/5	0/5	0/5	0/5
45 min occlusion, 4h recovery	5/5	5/5	1/5	1/5	1/5	0/5	0/5	0/5
45 min occlusion, 24h recovery	5/5	5/5	4/5	4/5	1/5	0/5	5/5	0/5
60 min occlusion, 4h recovery	7/7	7/7	3/5	3/5	3/5	0/5	4/5	1/5
60 min occlusion, 24h recovery	5/5	5/5	1/5	1/5	1/5	0/5	4/5	0/5
Koizumi Thick, 4h recovery	6/6	6/6	3/6	2/6	2/6	0/6	2/6	1/6
Longa Thick, 4h recovery	5/5	5/5	1/5	1/5	1/5	0/5	1/5	0/5

Koizumi Short, 4h recovery	5/5	5/5	1/5	2/5	2/5	0/5	1/5	0/5
Longa Short, 4h recovery	5/5	5/5	2/5	2/5	1/5	0/5	1/5	0/5
